# Inducing Nucleophilic Reactivity at Beryllium with an Aluminyl Ligand

**DOI:** 10.1021/jacs.3c00480

**Published:** 2023-02-14

**Authors:** Josef T. Boronski, Lewis R. Thomas-Hargreaves, Mathias A. Ellwanger, Agamemnon E. Crumpton, Jamie Hicks, Deniz F. Bekiş, Simon Aldridge, Magnus R. Buchner

**Affiliations:** † Chemistry Research Laboratory, Department of Chemistry, University of Oxford, Oxford, OX1 3TA, United Kingdom*;*; ‡ Fachbereich Chemie, Philipps-Universität Marburg, Marburg 35037, Germany

## Abstract

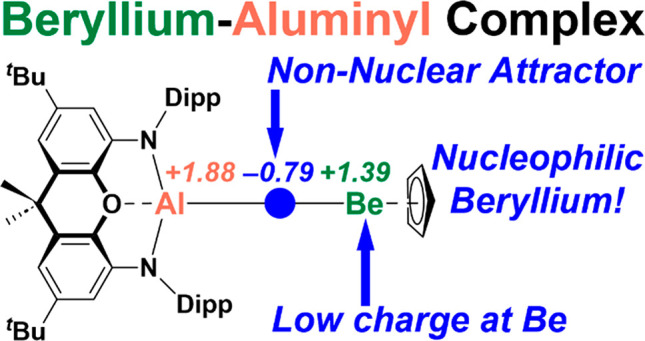

The reactions of anionic aluminium or gallium nucleophiles {K[E(NON)]}_2_ (E = Al, **1**; Ga, **2**; NON = 4,5-bis(2,6-diisopropylanilido)-2,7-di*tert*-butyl-9,9-dimethylxanthene) with beryllocene (BeCp_2_) led to the displacement of one cyclopentadienyl ligand at beryllium and the formation of compounds containing Be–Al or Be–Ga bonds (NON)EBeCp (E = Al, **3**; Ga, **4**). The Be–Al bond in the beryllium–aluminyl complex [2.310(4) Å] is much shorter than that found in the small number of previous examples [2.368(2) to 2.432(6) Å], and quantum chemical calculations suggest the existence of a non-nuclear attractor (NNA) for the Be–Al interaction. This represents the first example of a NNA for a heteroatomic interaction in an isolated molecular complex. As a result of this unusual electronic structure and the similarity in the Pauling electronegativities of beryllium and aluminium, the charge at the beryllium center (+1.39) in **3** is calculated to be less positive than that of the aluminium center (+1.88). This calculated charge distribution suggests the possibility for nucleophilic behavior at beryllium and correlates with the observed reactivity of the beryllium–aluminyl complex with *N,N′*-diisopropylcarbodiimide—the electrophilic carbon center of the carbodiimide undergoes nucleophilic attack by beryllium, thereby yielding a beryllium–diaminocarbene complex.

The diagonally related elements, beryllium and aluminium, have near identical Pauling electronegativity (1.57 and 1.61, respectively).^1,2^ Both of these elements are readily oxidized to their respective group oxidation state, in each case forming an ion (Be^2+^ or Al^3+^) with exceptional charge density (6.45 and 6.00 Å^–1^, respectively).^1c^ These ions are the two hardest main group Lewis acids and, hence, the chemistries of beryllium and aluminium are dominated by their behavior as electrophiles.^1,2^ Notably, the chemistry of beryllium is perhaps the least developed of all nonradioactive elements.^1^ This is because, principally, of the health hazards associated with beryllium and its compounds.^3^

Nevertheless, in recent years progress has been made in the preparation of novel beryllium-containing molecules, including compounds with unusual bonding motifs and/or electronic structures (Figure 1).^4^ For example, compounds with covalent bonds between beryllium and electropositive elements, such as aluminium and boron, have been reported, as have compounds that feature Be=C and Be=N double bonds (Figure 1A–C).^5−7^ Complexes that can even be considered to contain the metal in the 0 or +1 oxidation state have been reported (Figure 1D,E), although alternative descriptions of these systems have also been proposed.^8^ Indeed, conclusive evidence of these compounds reacting as sources of low-valent beryllium has yet to be reported.^8d,9^

**Figure 1:**
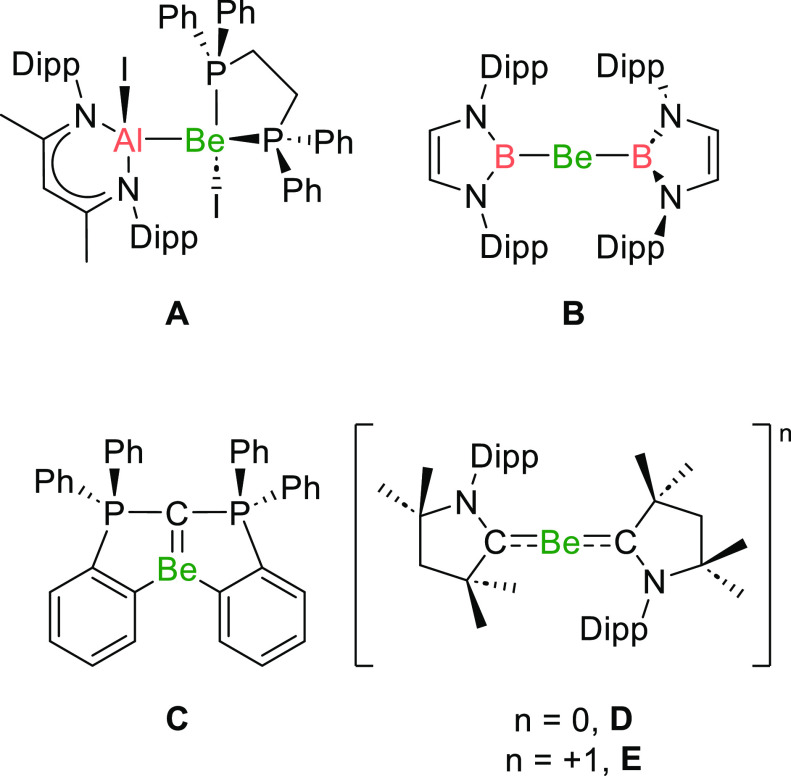
Notable recently reported beryllium-containing compounds.

Contrastingly, the reactivity of low-valent aluminium compounds has recently become an intensively investigated topic in main group chemistry.^10^ Within this surge of activity was a report in 2018 of a nucleophilic aluminyl anion (along with its gallium analogue) {K[E(NON)]}_2_ (E = Al, **1**; Ga, **2**; NON = 4,5-bis(2,6-diisopropylanilido)-2,7-di*tert*-butyl-9,9-dimethylxanthene).^11^ The potent nucleophilicity of compound **1** (and related aluminyl systems) has been harnessed to prepare a range of complexes featuring aluminium–metal bonds, many of which display unique behavior because of the powerful electron-donating properties of this aluminyl “metalloligand.”^11−15^

Here, we report the synthesis of a new beryllium–aluminyl complex.^6,16^ Atoms in molecules (QTAIM) calculations indicate that the Be–Al interaction features a non-nuclear attractor (NNA)—that is, a non-nuclear maximum in the electron density.^4,12a,17−19^ No example of this phenomenon in a heteronuclear metal–metal-bonded molecule has yet been reported. QTAIM calculations indicate a lower charge at beryllium than aluminium in this compound. Consequently, the beryllium–aluminyl complex is found to display behavior expected of a beryllium-centered nucleophile or a low-valent beryllium compound—a first example of such reactivity for beryllium.^8,19^ Contrastingly, the corresponding beryllium–gallyl complex does not have a NNA and is calculated to feature a higher positive charge at beryllium than gallium. Consequently, it displays divergent reactivity from its aluminium counterpart.

As **1** and **2** have already proved useful in the synthesis of a range of M–E bonds, their reactions with beryllocene BeCp_2_ were examined. It was envisaged that the “slipped” (η^1^-C_5_H_5_) ligand in beryllocene might be readily displaced by a stronger nucleophile.^21^ The reactions of BeCp_2_ with one equivalent of **1** or **2** lead to the precipitation of KCp and the formation of the new beryllium complexes (NON)EBeCp (E = Al, **3**; Ga, **4**) in 80% (**3**) or 58% (**4**) isolated yields (Scheme 1). Complex **3** represents the fourth example of a complex with a Be–Al bond, and **4** is the first with a Be–Ga bond.^6,16,21−23^

**Scheme 1:**
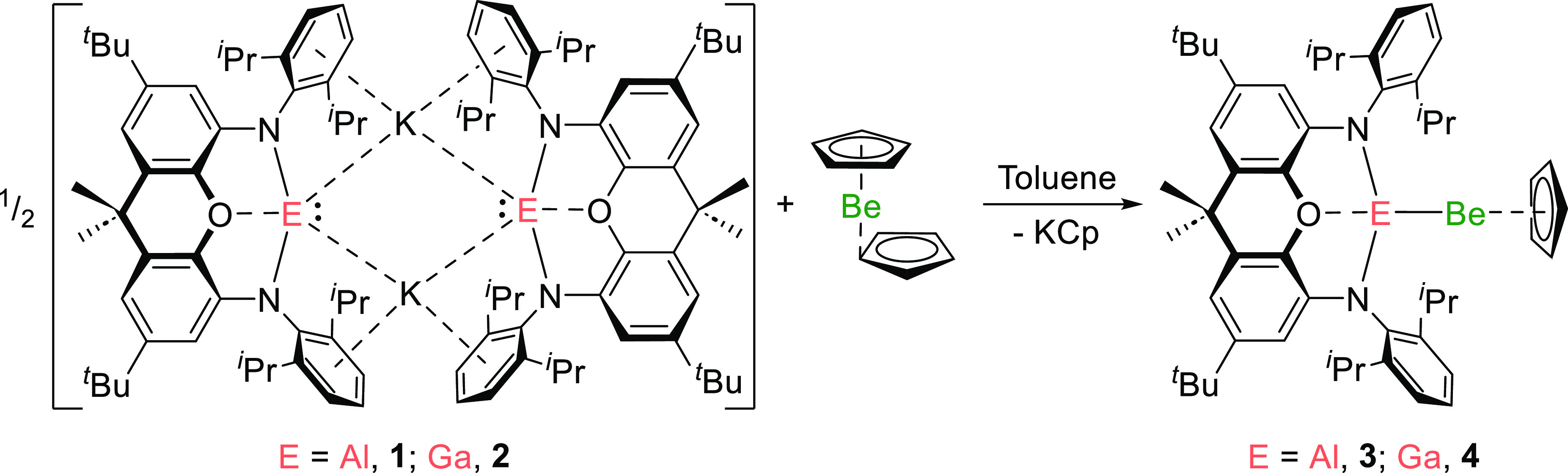
Synthesis of Beryllium–Aluminyl (3) and −Gallyl (4) Complexes

The molecular structure of **3** and **4** was confirmed in each case by single-crystal X-ray diffraction (Figures 2 and S10).^23^ The Be–Al distance in **3** [2.310(4) Å] is significantly shorter than those measured for the three previous examples of this type of bond: [(^Dipp^Nacnac)(I)AlBe(I)(dppe)] (**A**), 2.368(2) Å; [(^Dipp^Nacnac)(Br)AlBe(Br)(tmeda)] (**F**), 2.431(6) Å; and [(^Dipp^Nacnac)(I)AlBe(I)(tmeda)] (**G**), 2.432(6) Å {tmeda = *N,N,N′,N′*-tetramethylethylenediamine; ^Dipp^Nacnac = [(DippNCMe)_2_CH]^−^, Dipp = 2,6-diisopropylphenyl; and dppe = bis(diphenylphosphino)ethane}.^6,16^ Complexes **A**, **F**, and **G** were prepared via insertion of the neutral aluminylene Al(^Dipp^Nacnac) into beryllium-halogen bonds, rather than the metathesis approach employed for **3** and **4**.^6,16^

**Figure 2:**
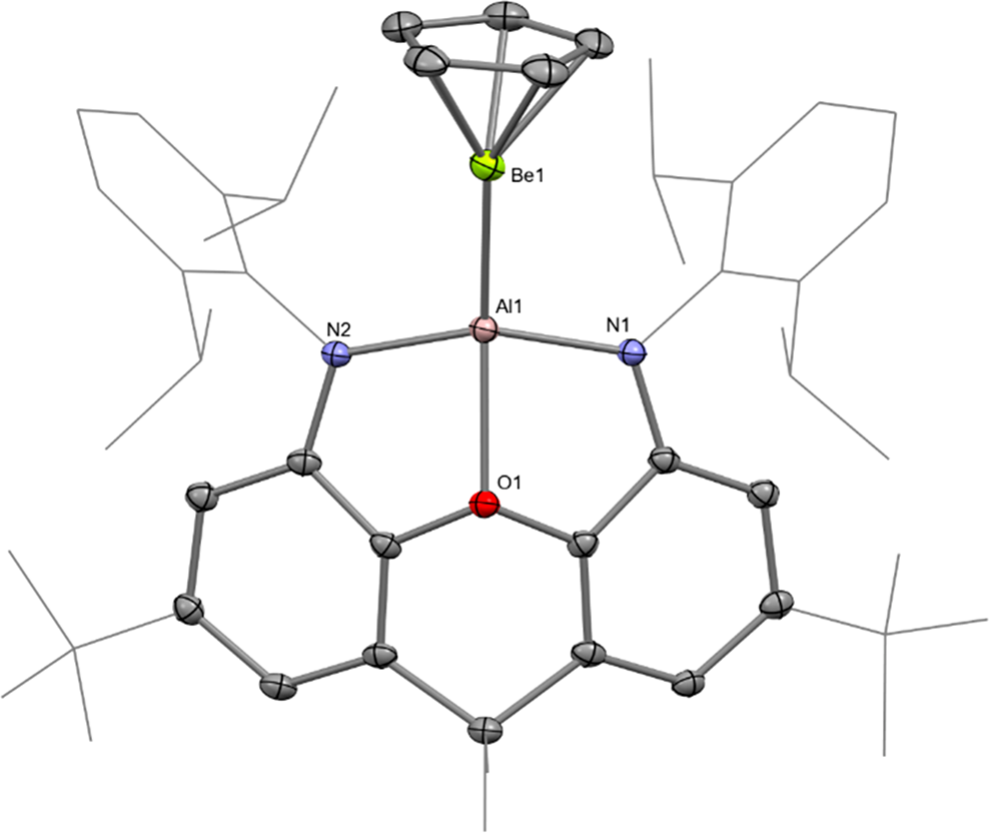
Molecular structure of **3** in the solid state, as determined by X-ray crystallography. Thermal ellipsoids set at 50% probability.

The shortness of the Be–Al bond in **3** is likely due to the greater σ-donor strength of the [(NON)Al]^−^ fragment compared with the [(X)Al(^Dipp^Nacnac)]^−^ unit within **A**, **F**, and **G**.^12^ The Be–Ga distance of 2.206(2) Å measured for **4** has no precedent in the literature—it was reported that Ga(^Dipp^Nacnac) does not react with BeX_2_(tmeda) (X = Br, I).^6^ Indeed, gallium is only the third metal to which beryllium has been bonded in a molecular complex, after platinum and aluminium.^6,16,20^ Both the Be–Al distance in **3** and the Be–Ga separation in **4** are in reasonable agreement with the sum of the single-bond covalent radii for the respective elements (2.28 and 2.26 Å, respectively).^24^

The Be-(η^5^-C_5_) centroid distances in **3** and **4** [1.498(2) and 1.501(2) Å, respectively] are lengthened compared with in BeCp_2_ [1.485(2) Å].^25^ The elongation of the Be-(η^5^-C_5_) centroid interaction in **3** and **4** vs BeCp_2_ could be viewed as the result of the (partial) reduction of the beryllium center. This is particularly relevant for **3**, given the similarity in the Pauling electronegativity of Be (1.57) and Al (1.61) for which an aluminium(II)/beryllium(I) formalism could be ascribed, thereby emphasizing the covalent nature of the Be–Al bond.^4,6,16^ However, these oxidation state formalisms are only useful insofar as they provide a predictor of the reactivity of a particular metal center.

The electronic structures of compounds **3** and **4** were probed via quantum theory of atoms in molecules (QTAIM) and natural bond orbital (NBO) calculations; the previously reported complex [(^Dipp^Nacnac)(Br)AlBe(Br)(tmeda)] (**F**) was also probed for comparative purposes (B3LYP D3BJ def2-TZVP def2/J). In the case of **3**, the Wiberg bond index (WBI) for the Be–Al interaction (0.82) is somewhat greater than the WBIs for the Be–Ga bond within **4** (0.73) and the Be–Al bond within **F** (0.64). Natural atomic orbital (NAO) analysis of **3** indicates significant population of the beryllium 2s orbital (0.73 e). This is a much greater degree of population than in the case of **4** (0.63 e) or **F** (0.57 e). Additionally, NBO analysis suggests that the Be–Al interaction in **3** comprises 37% Be (2s, 92%; 2p, 7.6%) and 63% Al (3s, 66%; 3p, 33%) character. The contribution from Be to the Be–Al bond is much lower in **F** (26%). Therefore, the WBI, NAO, and NBO data cumulatively suggest a significantly greater degree of covalency to the Be–Al interaction in **3** than the Be–E bonds in **4** (E = Ga) and **F** (E = Al).^6^ This also perhaps helps rationalize why the crystallographically determined Be–Al distance in **3** is significantly shorter than within **F**.

Topological analysis of **3** reveals a (3, −3) critical point—or a non-nuclear attractor (NNA)—between the aluminium and beryllium centers (Figure 3).^17^ A non-nuclear attractor is a local maximum in electron density that is not associated with the nucleus of an atom. Such critical points have been found for the Mg–Mg bond of the magnesium(I) compound [(DippNacnac)Mg]_2_ (**H**) and the Al–Al bonds of a number of dialanes {including [(NON)Al]_2_ (**I**)} within metallic beryllium and have been hypothesized for the Be–Be interaction in the (unknown) beryllium(I) complex diberyllocene (CpBe)_2_ (**J**).^12a,17−19,26^ Topologically, the electron density in the Be–Al internuclear region has a very flat profile that closely resembles that calculated for **H**, **I**, and **J** (Figure 3a).^12a,17,26^ In contrast to **3**, beryllium–gallyl complex **4** is not calculated to feature a NNA (Figures S19 and S20).

**Figure 3:**
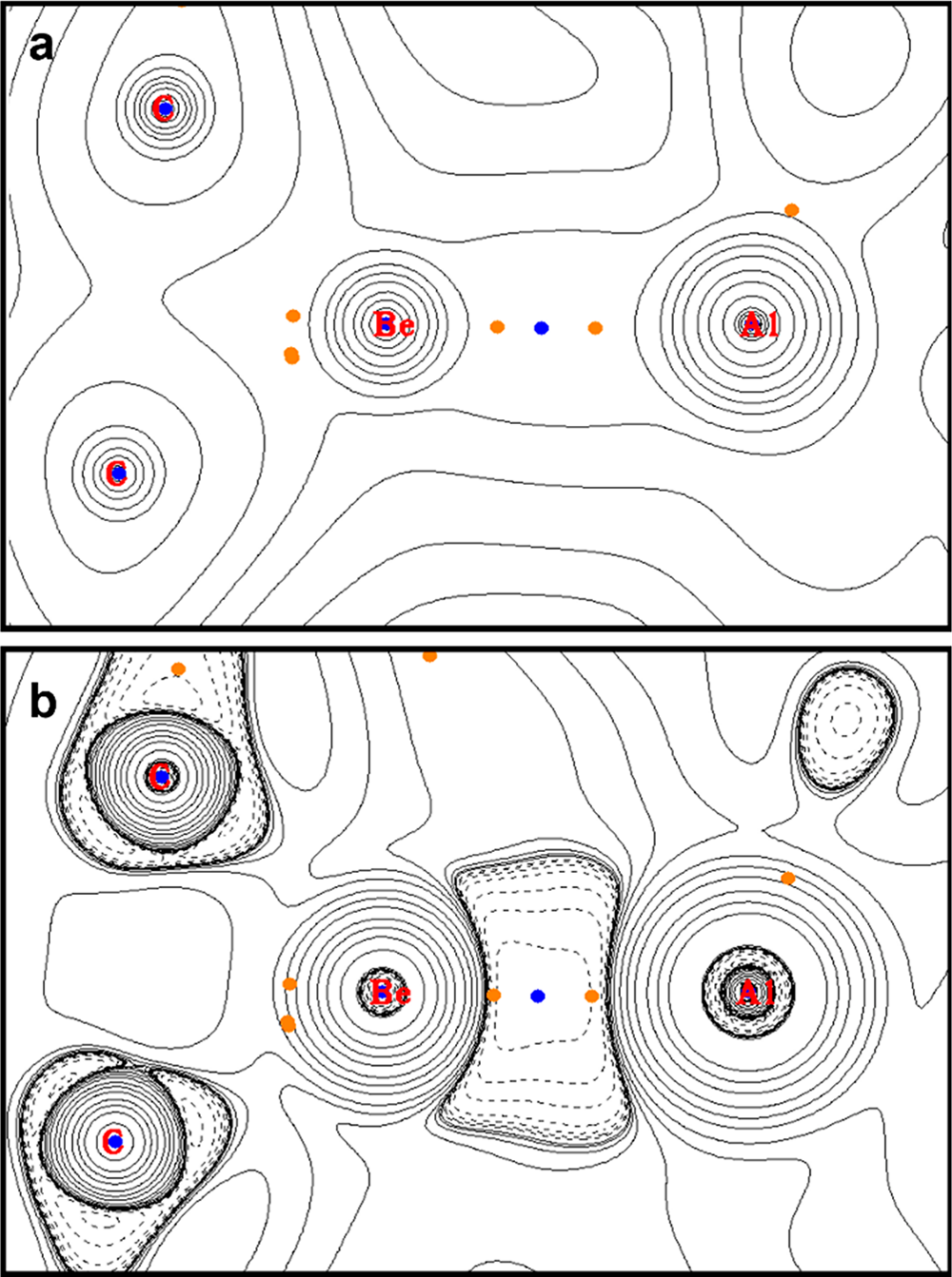
Topological QTAIM-derived plot of ρ(*r*) (a) and ∇^2^ ρ(*r*) (b) for **3**. Orange points are BCPs; blue points are (3, – 3) points/NNAs.

Because of the presence of the NNA within **3**, the charges distribution in this complex is very different from that calculated for **4** (Figure S21 and S22). In the case of **3**, QTAIM basin analysis yields charges of +1.88, +1.39, and −0.79 for Al, Be, and the NNA, respectively, which implies that the partial positive charge at Be is lower than at Al. Therefore, this picture of the charge distribution in **3** contrasts with those calculated for **A**, **F**, and **G**, for which the partial positive charge at Be is greater than at Al.^6,16^ Most interestingly, these data hint at the possibility for nucleophilic reactivity from the beryllium center in **3** and further evidence an aluminium(II)/beryllium(I) assignment for this compound.^4,13a^ Within **4**, the QTAIM charge at Ga is calculated to be +0.61, and the charge at Be is +1.49. Hence, the polarity of the Be–Ga bond in **4** is calculated to be opposite that of the Be–Al bond in **3**. This is also supported by the ^9^Be NMR spectroscopic parameters of these two compounds, as discussed in the ESI (Figure S12).^22^ As a result, contrasting reactivity would be expected from **3** and **4**.

To examine whether **3** might act as a source of nucleophilic beryllium, the reactivity of this complex with *N,N′*-diisopropylcarbodiimide was examined (Scheme 2, right). Reactions with carbodiimides have previously been used as an experimental probe of the polarity of Al–Au, −Ag, −Cu, and −Zn bonds, among others.^12a,13^ Interestingly, complex **3** reacts rapidly and quantitatively with a single equivalent of this substrate. Crystallization of the reaction product, (NON)Al{(N^*i*^Pr)_2_C}BeCp (**5**), from hexane allowed for determination of the connectivity within this molecule via X-ray diffraction (Figure 4). Accordingly, complex **5** is formed via insertion of the carbodiimide into the Be–Al bond of **3** with accompanying reduction and conversion of the heteroallene into a bent diaminocarbene.^13a^

**Scheme 2:**
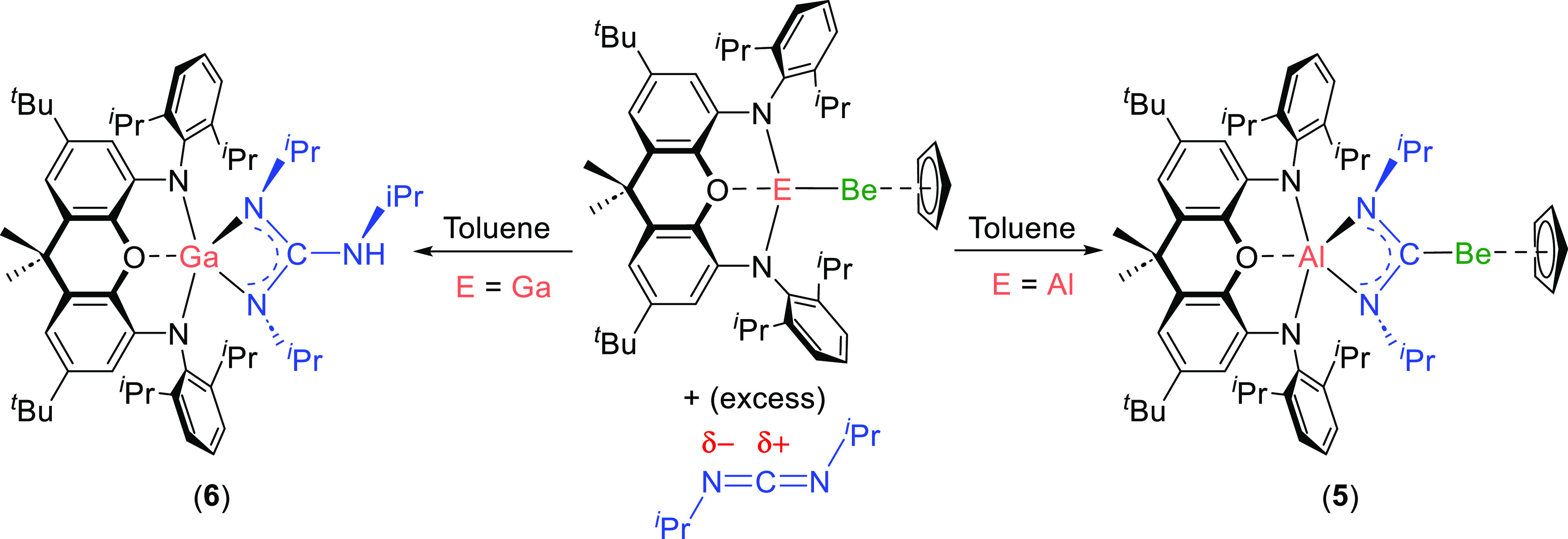
Reactivity of **3** and **4** with Excess *N,N*′-Diisopropylcarbodiimide

**Figure 4:**
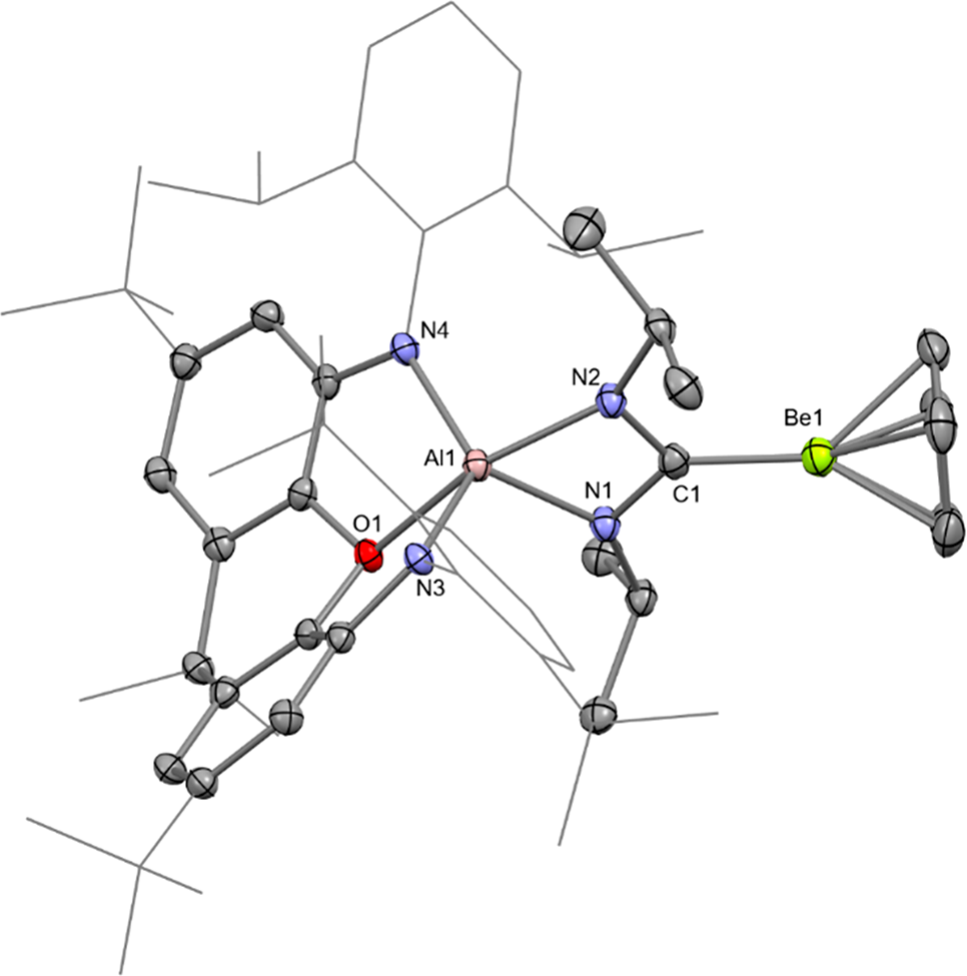
Molecular structure of **5** in the solid state, as determined by X-ray crystallography. Thermal ellipsoids set at 50% probability.

Aluminium is chelated by the nitrogen centers within the resulting metalla-amidinate moiety of **5**, with the carbenic center coordinating to beryllium. This connectivity is consistent with nucleophilic attack by beryllium at the electrophilic carbon center of the carbodiimide, while the aluminium center acts as the electrophilic site, which results in Al–N bond formation. Although steric factors may influence the product formed in this reaction, the result is in line with the calculated charge distribution within **3**.^27^ The nucleophilic reactivity of s-block metals is extremely rare, and previously unknown for beryllium.^19^

The reactivity of **4**—for which the polarity of the M–M bond is calculated to be inverted compared with **3**—with *N,N′*-diisopropylcarbodiimide was also examined (Scheme 2, left). In this case, NMR spectroscopy indicates that the reaction leads to the formation of BeCp_2_ and isopropyl isocyanide, in addition to a new gallium-containing product. Crystallization from hexane yields orange crystals of the monometallic gallium complex (NON)Ga{(N^*i*^Pr)_2_C(NH^*i*^Pr)} (**6**), which was structurally characterized by X-ray crystallography (Figure S11). Complex **6** features a guanidinate-type ligand coordinated to gallium, formed by the combination of two carbodiimide units. Pertinently, a very similar product has been reported for the reaction of the sodium–gallyl complex (^Dipp^BIAN)GaNa(dme)_2_ {^Dipp^BIAN = 1,2-bis[(2,6-diisopropylphenyl)imino]acenaphthene; dme = 1,2-dimethoxyethene} with *N,N′*-dicyclohexylcarbodiimide (Figure 5ii).^28^ In this case, gallium is proposed to act as a nucleophile, which couples carbodiimide to guanidinate, with the sodium cation acting as the electrophile (Figure 5).^28^ Additionally, crystallographic evidence suggests that the reaction progresses via the formation of a metalla-amidinate intermediate with the carbenic center bonded to gallium, and the nitrogen atoms bonded to sodium (Figure 5i). This evidence, and the structural characterization of **6**, suggests that gallium acts as the nucleophile in the reaction of **4** with the carbodiimide, with beryllium acting as the electrophile.

**Figure 5:**
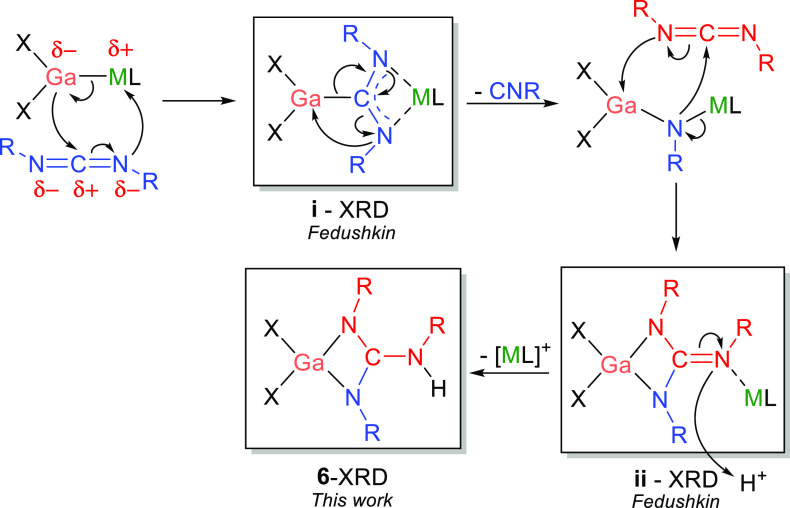
Possible mechanism for the formation of **6**. X = amide group [NR_2_]^−^ ; L = dme (M = Na) or Cp^–^ (M = Be).

In summary, beryllium–aluminyl and −gallyl complexes have been prepared via metathesis reactions. Quantum chemical calculations indicate the existence of a non-nuclear attractor for the Be–Al interaction of **3**. This unusual electronic structure and the similar Pauling electronegativities of beryllium and aluminium result in a calculated charge at the beryllium center, which is lower than that at aluminium. Consistently, the result of the reaction between **3** and *N,N*′-diisopropylcarbodiimide reveals that beryllium acts as a nucleophile by attacking the electrophilic carbon center of the carbodiimide.

## Supporting Information Available

Synthesis details, NMR spectra, and crystallographic and computational details (PDF)
