# Supplementary material for: Inducing Nucleophilic Reactivity at Beryllium with an Aluminyl Ligand
Source: J Am Chem Soc. 2023 Feb 14;145(8):4408–13. doi: 10.1021/jacs.3c00480 (PMC9983009; doi:10.1021/jacs.3c00480)
Supplement: Supplementary file 1 — ja3c00480_si_001.pdf [file ja3c00480_si_001.pdf]

Electronic Supporting Information

Inducing Nucleophilic Reactivity at Beryllium with an Aluminyl Ligand

Josef T. Boronski[a*], Lewis R. Thomas-Hargreaves[b], Mathias A. Ellwanger[a], Agamemnon E. Crumpton[a], Jamie Hicks[a], Deniz F. Bekiş[b], Simon Aldridge[a*], Magnus R. Buchner[b*]
[a]) Chemistry Research Laboratory, Department of Chemistry, Oxford, OX1 3TA, U.K.
[b]) Fachbereich Chemie, Philipps-Universität Marburg, Marburg 35043, D.E.

Contents

General Considerations – S2

Synthesis of Novel Compound – S2

Spectroscopic Data – S5

Crystallographic Data – S10

Discussion of ^{9}Be NMR Chemical Shifts – S14

Computational Details – S15
Discussion of Molecular Orbitals Calculated for **3** and **4** – S16
Discussion of Charge Distribution and Electron Topology Calculated for **3** and **4** – S20

References – S25

Experimental Details

General Considerations

All manipulations were carried out using Schlenk line or glovebox techniques under an atmosphere of argon or dinitrogen. Solvents were dried by passage through activated alumina towers, dried with NaK_2 and degassed before use. Solvents were stored over NaK_2. NMR spectra were measured in C_6D_6 which was dried over NaK_2, with the solvent being distilled under reduced pressure, degassed by three freeze-pump-thaw-cycles and stored under argon in a Teflon valve ampoule. NMR samples were prepared under argon in 5 mm Wilmad 507-PP tubes fitted with J. Young Teflon valves. NMR spectra were measured on a Bruker Avance III HD Nanobay 400 MHz NMR spectrometer equipped with a 9.4 T magnet, Bruker Avance III 500 MHz NMR spectrometer equipped with a 11.75 T magnet, or a Bruker Avance III NMR 500 MHz NMR spectrometer equipped with a 11.75 T magnet and a ^{13}C detect cryoprobe. ^{1}H and ^{13}C NMR spectra were referenced internally to residual protio-solvent (^{1}H) or solvent (^{13}C) resonances and are reported relative to tetramethylsilane (δ = 0 ppm). ^{9}Be NMR spectra were referenced to a 0.43 M solution of $BeSO_4 \cdot 4H_2O$ in D_2O (δ = 0 ppm). Chemical shifts are quoted in δ (ppm) and coupling constants in Hz. FTIR spectra were recorded on a Bruker Alpha spectrometer with Platinum-ATR module. Elemental analyses were carried out by London Metropolitan University. The compounds $[K\{Al(NON)\}]_2$ (**1**),[1] $[K\{Ga(NON)\}]_2$ (**2**),[1] and $BeCp_2$ were prepared as described previously.[2] *N,N'*-diisopropylcarbodiimide (CDI) was dried over 3 Å molecular sieves, degassed, distilled, and stored over 3 Å molecular sieves.

Synthesis of Novel Compounds

Synthesis of (NON)AlBeCp (3)

To an ampoule fitted with a Teflon valve and equipped with a glass-coated stirrer bar was added a solid mixture of **1** (100 mg, 0.136 mmol) and $BeCp_2$ (19 mg, 0.137 mmol, 1.01 equ.). Toluene (7 mL) was condensed into the vessel *in vacuo* at –196 °C. The pale-yellow solution was allowed to warm to room temperature and stirred overnight, leading to the formation of a colourless precipitate. The resulting suspension was filtered and volatiles were removed *in vacuo*. The waxy off-white solid was dried *in vacuo* for a further two hours at 60 °C to remove unreacted $BeCp_2$. Subsequently, the solid was washed with cold (–78 °C) hexane (2 x 1 mL) and dried *in vacuo*, yielding an off-white powder. Yield: 84 mg, 80%. Single crystals of **3** suitable for X-ray diffraction experiments were obtained by storage of a benzene solution in an NMR tube for several days. Compound **3** is rather sensitive, even in the solid state and under an inert atmosphere, and completely decomposes over the course of one month at room temperature. Anal. Calcd for $C_{52}H_{67}AlBeN_2O$: C, 80.89; H, 8.75; N, 3.63. Found: C, 80.66; H, 8.67; N, 3.42. ^{1}H NMR (400 MHz, C_6D_6, 298 K): δ = 1.15 (d, $^{3}J_{HH}$= 7.0 Hz, 12H, $CH(CH_3)_2$), 1.23 (s, 18H, $C(CH_3)_3$), 1.27 (d, $^{3}J_{HH}$= 7.0 Hz, 12H, $CH(CH_3)_2$), 1.56 (s, 6H, $C(CH_3)_2$),

3.45 (sept, $^3J_{HH}$= 7.0 Hz, 4H, C*H*(CH$_3$)$_2$), 5.15 (s, 5H, C$_5$*H*$_5$), 6.38 (d, $^4J_{HH}$= 1.9 Hz 2H, XA-*o*-C*H*), 6.75 (d, $^4J_{HH}$= 1.9 Hz 2H, XA-*p*-C*H*), 7.26 (m, 6H, Ar*H*); ^{13}C{^{1}H} NMR (101 MHz, C$_6$D$_6$): δ = 24.52, 25.65 (CH(*C*H$_3$)$_2$), 28.05 (*C*(CH$_3$)$_2$), 29.17(*C*H(CH$_3$)$_2$), 31.87 (C(*C*H$_3$)$_3$), 35.15 (*C*(CH$_3$)$_3$), 36.95 (C(*C*H$_3$)$_2$), 102.99 (*C*$_5$H$_5$), 107.27, 110.29, 123.94, 126.18, 132.70, 141.27, 141.85, 142.62, 148.00, 148.56 (Ar-*C*); ^{9}Be NMR (42 MHz, C$_6$D$_6$): δ = –28.77 ($w_{1/2}$ = 70.24 Hz).

Synthesis of (NON)GaBeCp (4)

To an ampoule fitted with a Teflon valve and equipped with a glass-coated stirrer bar was added a solid mixture of **2** (100 mg, 0.128 mmol) and BeCp$_2$ (18 mg, 0.130 mmol, 1.01 equ.). Toluene (7 mL) was condensed into the vessel *in vacuo* at –196 °C. The colourless solution was allowed to warm to room temperature and stirred overnight, leading to the formation of a colourless precipitate. The resulting suspension was filtered and volatiles were removed *in vacuo*. The waxy off-white solid was dried *in vacuo* for a further two hours at 60 °C to remove unreacted BeCp$_2$. Subsequently, the solid was extracted with hexane (3 x 10 mL) and the solution concentrated to 15 mL. Storage overnight at –30 °C yielded a crop of colourless crystals. Yield: 57 mg, 58%. Single crystals of **4** suitable for X-ray diffraction experiments were obtained by overnight evaporation of a concentrated hexane solution in a glovebox. Compound **4** is rather sensitive and shows signs of decomposition upon heating in the solid state and upon storage in solution at room temperature for 24 hours. Anal. Calcd for C$_{52}$H$_{67}$GaBeN$_2$O: C, 76.65; H, 8.29; N, 3.44. Found: C, 76.31; H, 8.10; N, 3.32. ^{1}H NMR (400 MHz, C$_6$D$_6$, 298 K): δ = 1.15 (d, $^3J_{HH}$= 6.8 Hz, 12H, CH(C*H*$_3$)$_2$), 1.16 (d, $^3J_{HH}$= 6.8 Hz, 12H, CH(C*H*$_3$)$_2$), 1.27 (s, 18H, C(C*H*$_3$)$_3$), 1.65 (s, 6H, C(C*H*$_3$)$_2$), 3.45 (sept, $^3J_{HH}$= 6.8 Hz, 4H, C*H*(CH$_3$)$_2$), 5.13 (s, 5H, C$_5$*H*$_5$), 6.38 (d, $^4J_{HH}$= 2.0 Hz 2H, XA-*o*-C*H*), 6.81 (d, $^4J_{HH}$= 2.0 Hz 2H, XA-*p*-C*H*), 7.23 (m, 6H, Ar*H*); ^{13}C{^{1}H} NMR (101 MHz, C$_6$D$_6$): δ = 24.77, 25.65 (CH(*C*H$_3$)$_2$), 27.71 (*C*(CH$_3$)$_2$), 28.71 (*C*H(CH$_3$)$_2$), 32.01 (C(*C*H$_3$)$_3$), 35.09 (*C*(CH$_3$)$_3$), 37.73 (C(*C*H$_3$)$_2$), 103.09 (*C*$_5$H$_5$), 107.40, 110.44, 123.90, 126.15, 134.42, 142.24, 142.43, 142.77, 147.00, 148.67 (Ar-*C*); ^{9}Be NMR (70 MHz, C$_6$D$_6$): δ = –26.92 ($w_{1/2}$ = 37.34 Hz).

Synthesis of (NON)Al{(N^{i}Pr)$_2$C}BeCp (5)

To an ampoule fitted with a Teflon valve and equipped with a glass-coated stirrer bar was added **3** (30 mg, 0.0389 mmol). Toluene (1 mL) and CDI (0.02 mL) were condensed into the vessel *in vacuo* at –196 °C. The pale yellow solution was allowed to warm to room temperature and stirred overnight. Subsequently, volatiles were removed *in vacuo*. The beige solid was dried *in vacuo* for a further two hours at 60 °C to remove unreacted CDI. Subsequently, the solid was extracted with hexane (3 x 3 mL) and the solution concentrated to 3 mL. Storage overnight at –30 °C yielded a crop of colourless crystals. Yield: 18 mg, 52%. Single crystals of **5** suitable for X-ray diffraction experiments were obtained by heating **5** into hexane and allowing the resulting solution to cool slowly to room temperature. Anal. Calcd for C$_{59}$H$_{81}$AlBeN$_4$O: C, 78.89; H, 9.09; N, 6.24. Found: C, 78.84; H, 9.00; N, 6.22. ^{1}H NMR (400 MHz, C$_6$D$_6$, 298 K): δ = 0.03 (d, $^3J_{HH}$ = 6.5 Hz, 6H, NCH(C*H*$_3$)$_2$), 1.02 (d, $^3J_{HH}$

= 6.8 Hz, 6H, ArCH(C*H*$_3$)$_2$), 1.08 (d, $^3J_{HH}$ = 6.7 Hz, 6H, ArCH(C*H*$_3$)$_2$), 1.18 (d, $^3J_{HH}$ = 6.5 Hz, 6H, NCH(C*H*$_3$)$_2$), 1.28 (m, 24H, ArCH(C*H*$_3$)$_2$ and C(C*H*$_3$)$_3$), 1.35 (d, $^3J_{HH}$ = 6.8 Hz, 6H, ArCH(C*H*$_3$)$_2$), 1.68 (s, 3H, C(C*H*$_3$)$_2$), 1.87 (s, 3H, C(C*H*$_3$)$_2$), 2.53 (sept, $^3J_{HH}$ = 6.5 Hz, 1H, NC*H*(CH$_3$)$_2$), 3.13 (sept, $^3J_{HH}$ = 6.5 Hz, 1H, NC*H*(CH$_3$)$_2$), 3.61 (sept, $^3J_{HH}$ = 6.8 Hz, 2H, ArC*H*(CH$_3$)$_2$), 3.73 (sept, $^3J_{HH}$ = 6.8 Hz, 2H, ArC*H*(CH$_3$)$_2$), 5.59 (s, 5H, C_5H_5), 6.11 (d, $^4J_{HH}$= 1.8 Hz 2H, XA-*o*-C*H*), 6.74 (d, $^4J_{HH}$= 1.8 Hz 2H, XA-*p*-C*H*), 7.32 (m, 6H, Ar*H*).; ^{13}C{^{1}H} NMR (101 MHz, C_6D_6): δ = δ 23.40 (C(*C*H$_3$)$_2$), 24.39 (NCH(*C*H$_3$)$_2$), 24.97 (ArCH(*C*H$_3$)$_2$), 25.72 (ArCH(*C*H$_3$)$_2$), 25.85 (NCH(*C*H$_3$)$_2$), 26.09 (ArCH(*C*H$_3$)$_2$), 26.46 (Ar*C*H(CH$_3$)$_2$), 26.86, 29.06 (Ar*C*H(CH$_3$)$_2$), 31.94 (C(*C*H$_3$)$_2$), 33.60, 35.16 (*C*(CH$_3$)$_2$), 37.03 (Ar*C*H(CH$_3$)$_2$), 49.43 (N*C*H(CH$_3$)$_2$), 50.60 (N*C*H(CH$_3$)$_2$), 104.42 (C_5H_5), 105.96, 111.16, 124.14, 124.33, 125.72, 131.66, 139.55, 144.95, 145.17, 147.27, 147.39, 148.21 (Ar-*C*), 200.88 (N*C*N).; ^{9}Be NMR (70 MHz, C_6D_6): δ = –24.56 ($w_{1/2}$ = 59.85 Hz).

Synthesis of (NON)Ga{(N^{i}Pr)$_2$C(NH^{i}Pr)} (6)

To an ampoule fitted with a Teflon valve and equipped with a glass-coated stirrer bar was added **4** (30 mg, 0.0389 mmol). Toluene (1 mL) and CDI (0.02 mL) were condensed into the vessel *in vacuo* at –196 °C. The pale orange solution was allowed to warm to room temperature and stirred overnight. Subsequently, volatiles were removed *in vacuo*. The waxy orange solid was dried *in vacuo* for a further two hours at 60 °C to remove unreacted CDI. Subsequently, the solid was extracted with hexane (3 x 3 mL) and the solution concentrated to 3 mL. Storage overnight at –30 °C yielded a crop of pale orange crystals. Yield: 18 mg, 53%. Single crystals of **6** suitable for X-ray diffraction experiments were obtained by allowing a concentrated hexane solution to stand for two hours at room temperature. Once isolated as crystalline material, **6** is insoluble in C_6D_6 and decomposes, with formation of a black/grey precipitate, upon heating. Therefore, NMR data of suitable quality could not be obtained for this compound. Anal. Calcd for $C_{63}H_{98}GaN_5O$: C, 74.83; H, 9.77; N, 6.93. Found: C, 74.77; H, 9.61; N, 6.83.

Spectroscopic Data

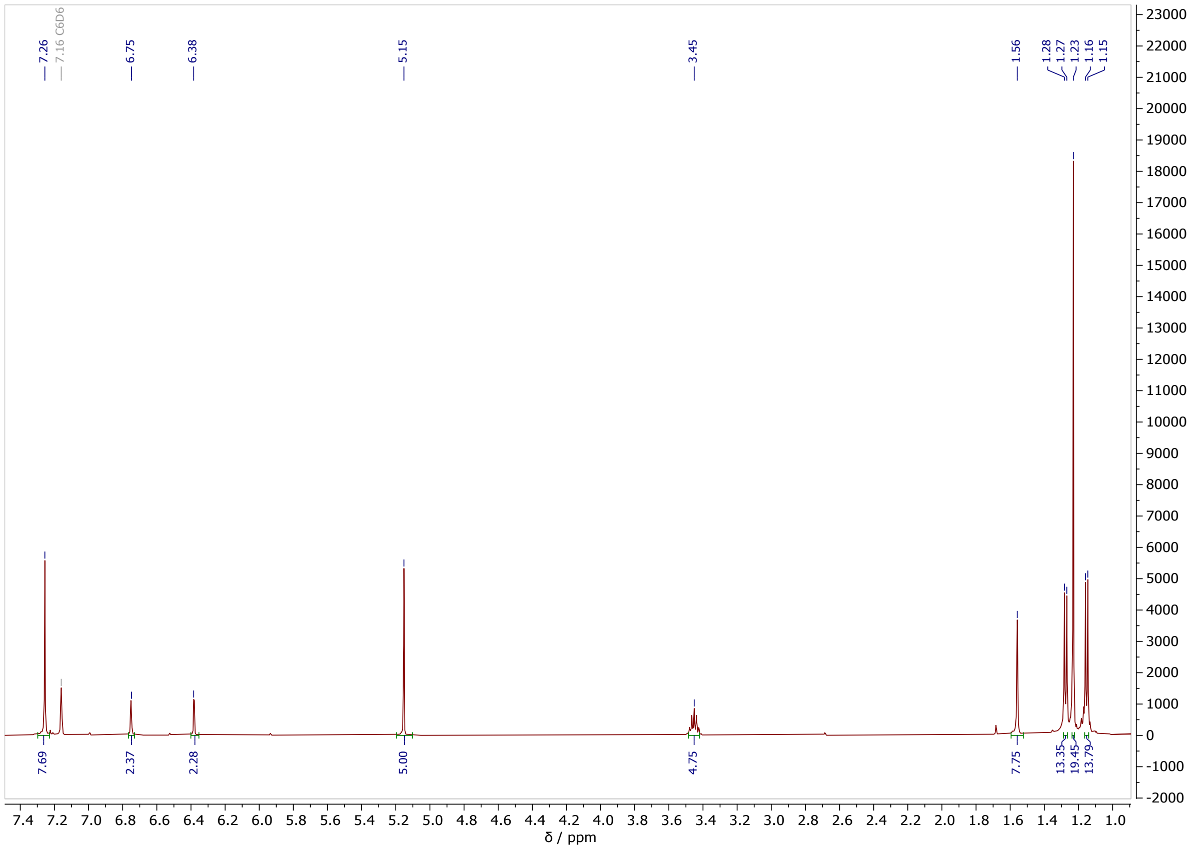

Figure S1: ^{1}H NMR spectrum of compound **3**.

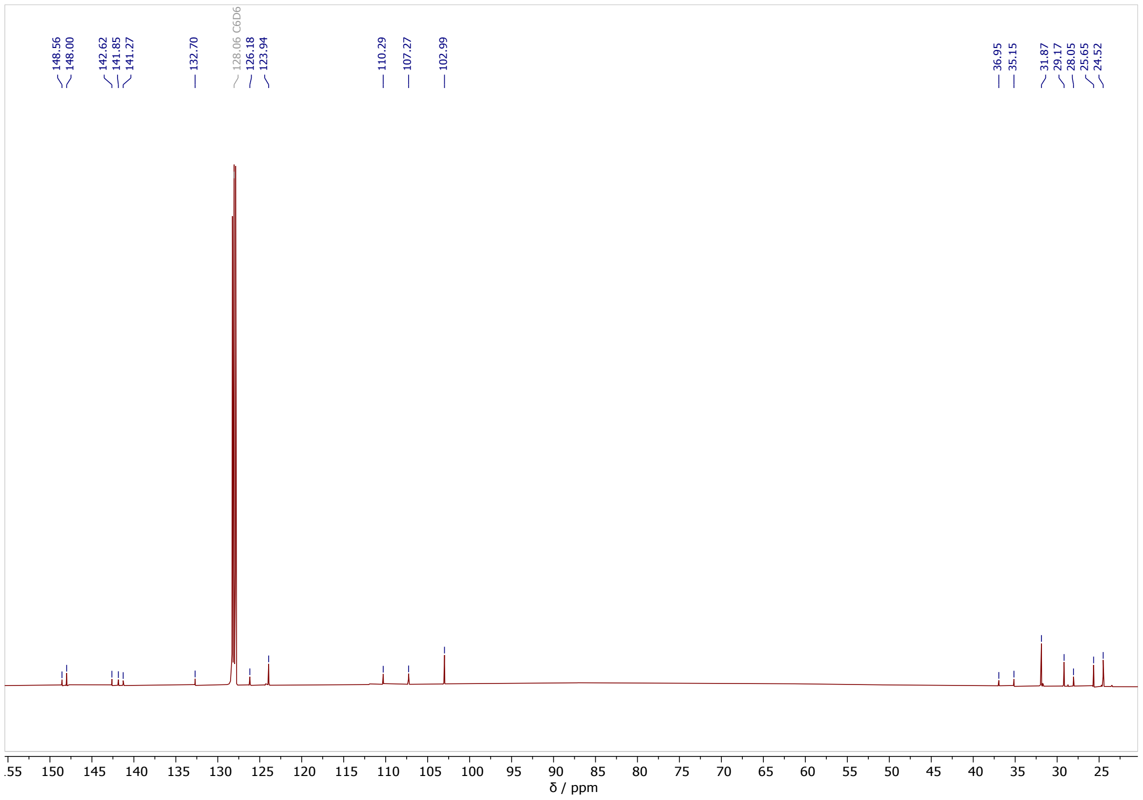

Figure S2: ^{13}C {^{1}H} NMR spectrum of compound **3**.

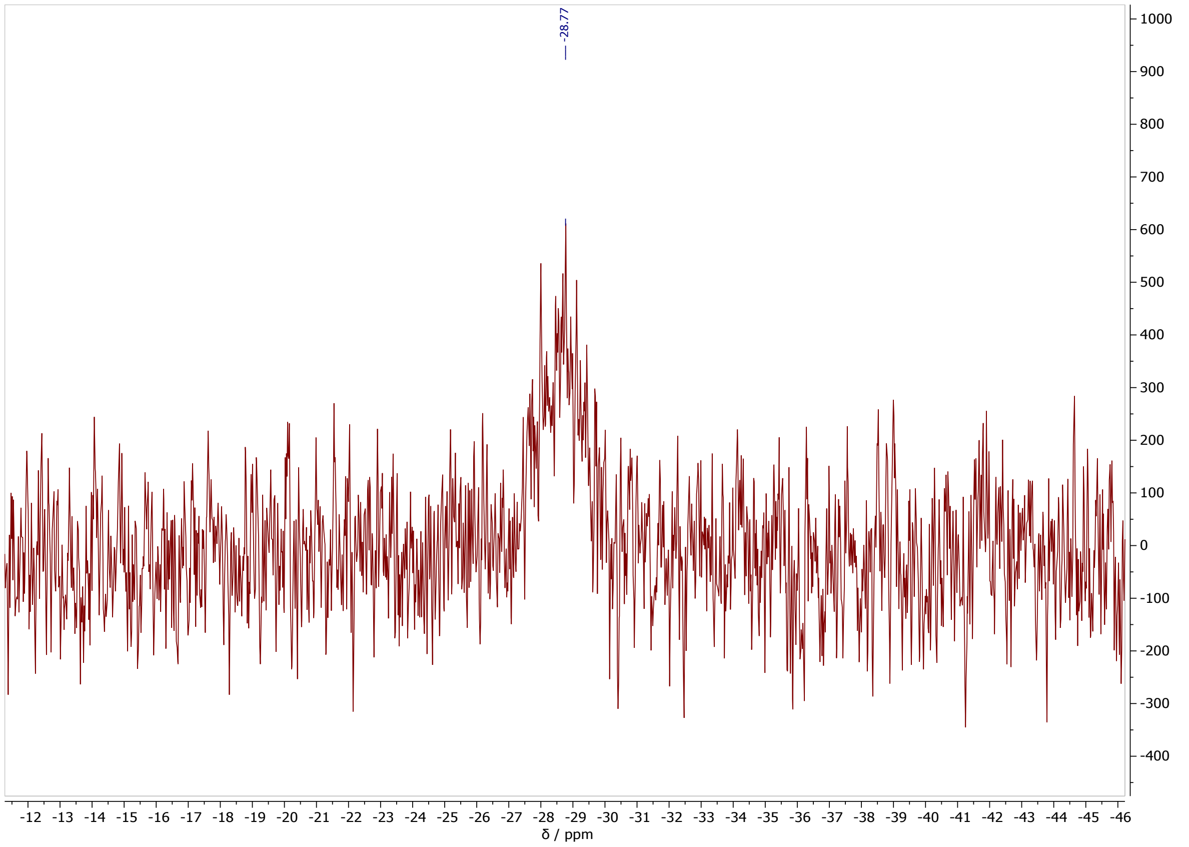

Figure S3: ^{9}Be NMR spectrum of compound **3**.

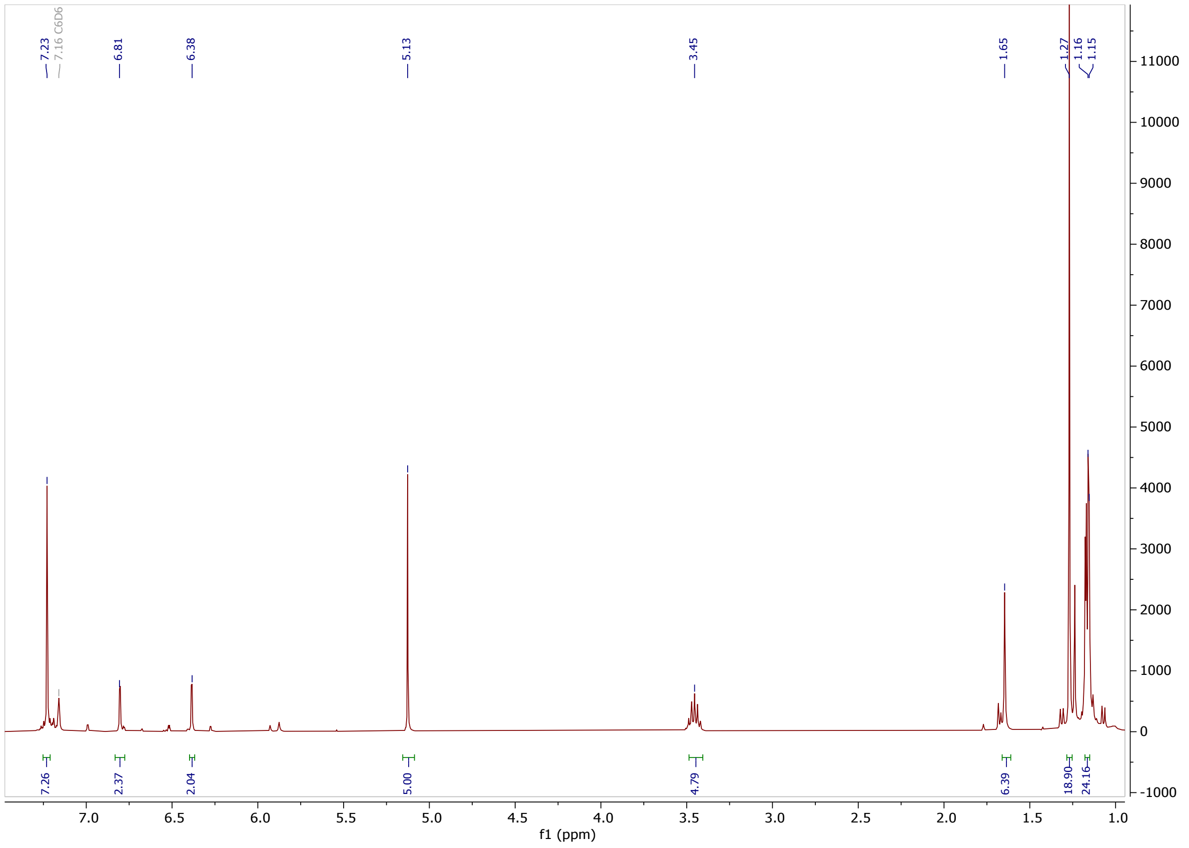

Figure S4: ^{1}H NMR spectrum of compound **4**.

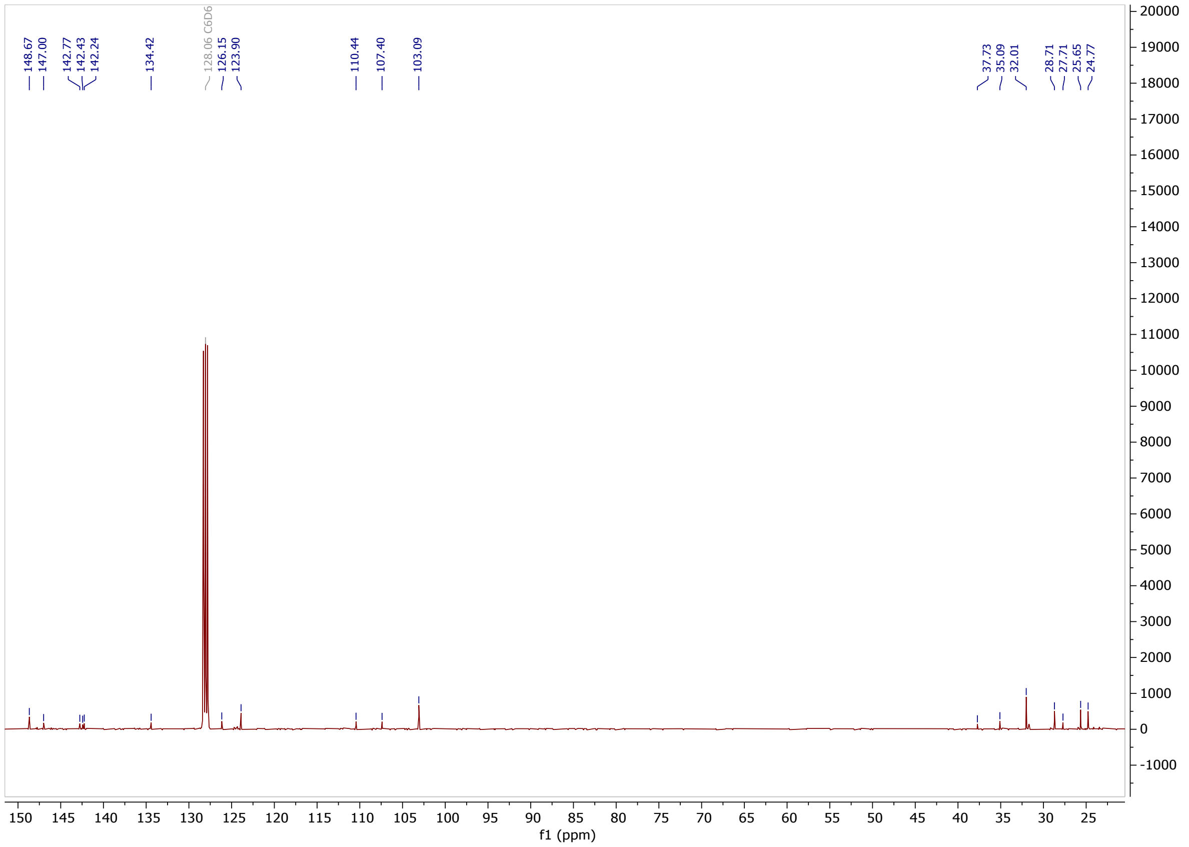

Figure S5: $^{13}C\ \{^{1}H\}$ NMR spectrum of compound **4**.

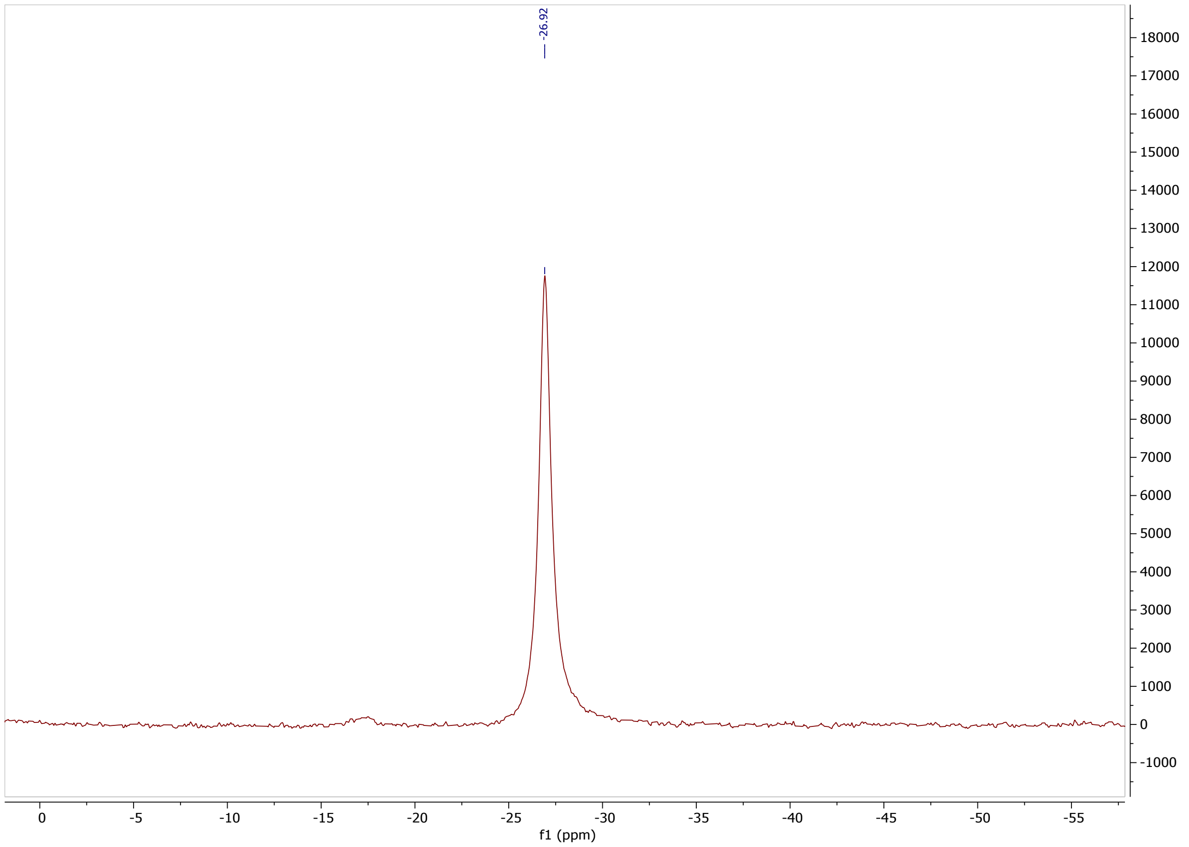

Figure S6: ^{9}Be NMR spectrum of compound **4**.

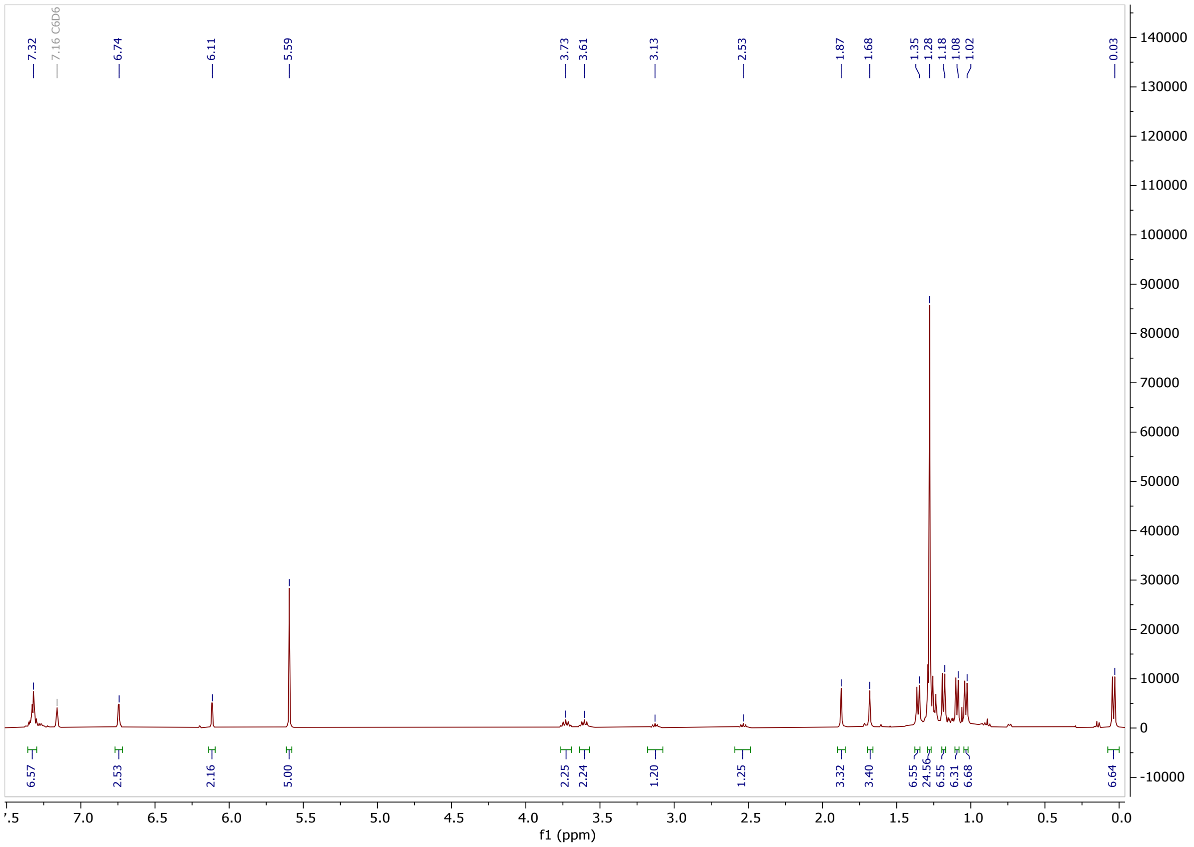

Figure S7: ^{1}H NMR spectrum of compound **5**.

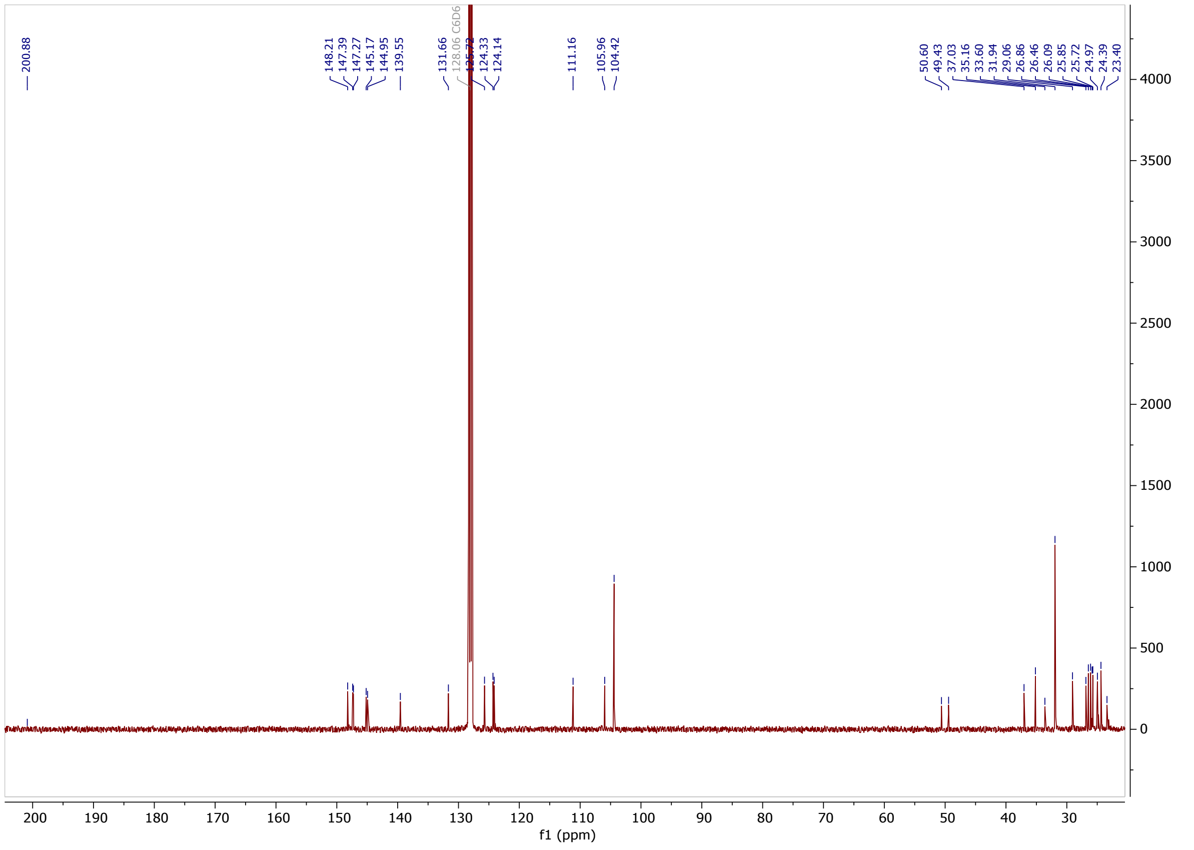

Figure S8: ^{13}C {^{1}H} NMR spectrum of compound **5**.

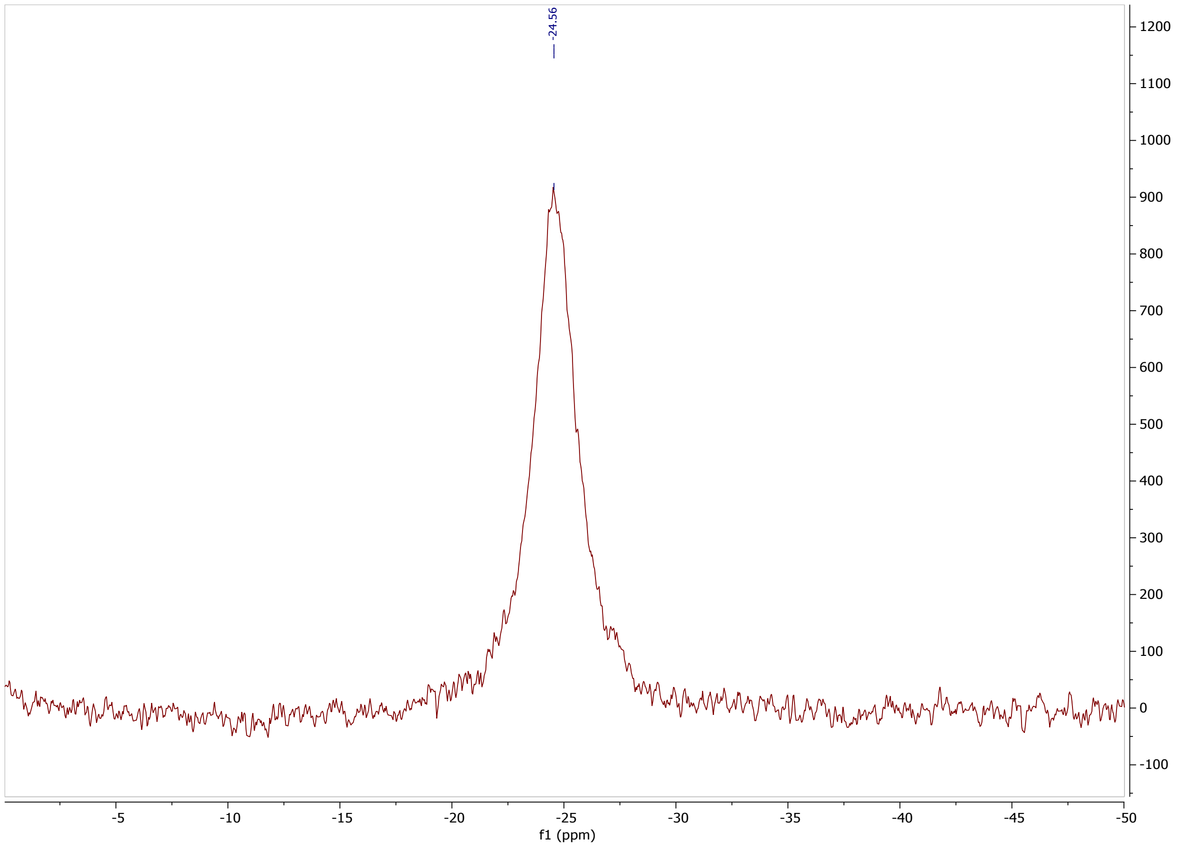

Figure S9: ^{9}Be NMR spectrum of compound **5**.

Crystallographic Data

Single-crystal X-ray diffraction data for compound **3** was collected on a Stoe Stadi Vari. Single crystals were selected under a pre-dried argon stream in perfluorinated polyether (Fomblin YR 1800, Solvay Solexis) and mounted using the MiTeGen MicroLoop system at ambient temperature. X-ray diffraction data was collected using the monochromated Cu-Kα (λ = 1.54186 Å) radiation of a Stoe StadiVari diffractometer equipped with a Xenocs Microfocus Source and a Dectris Pilatus 300 K. Evaluation, integration and reduction of the diffraction data was carried out using the X-AREA software suite.[15] Multi-scan absorption correction was applied with the LANA module of the X-AREA software suite. The structures were solved with dual-space methods (SHELXT-2018/2) and refined against F2 (SHELXL-2018/3) using the OLEX2 software package.[5-7] All atoms were located by Difference Fourier synthesis and non-hydrogen atoms refined anisotropically. Hydrogen atoms were refined using the “riding model” approach with isotropic displacement parameters 1.2 times of that of the preceding carbon atom. Data for **4**, **5**, and **6** were collected using an Oxford Diffraction/Agilent SuperNova or Rigaku XtaLAB Synergy-R. Crystals were selected under Paratone-N or perfluorinated oil, mounted on MiTeGen Micromount loops and quench-cooled using an Oxford Cryosystems open flow N_2 cooling device.[3] Selected details of data collection are given in Table 1. Data collected were processed using the CrysAlisPro package, including unit cell parameter refinement and inter-frame scaling (which was carried out using SCALE3 ABSPACK within CrysAlisPro).[4] Equivalent reflections were merged and diffraction patterns processed with the CrysAlisPro suite.[4] Structures were solved *ab initio* from the integrated intensities using SHELXT and refined on F2 using SHELXL with the graphical interface OLEX2.[5-7] Crystallographic data is given in the supplementary deposited CIF files (CCDC 2235387-2235390) and can be obtained free of charge from the Cambridge Crystallographic Data Centre *via* http://www.ccdc.cam.ac.uk/data_request/cif.

Table 1: Selected X-ray data collection and refinement parameters

	**3**	**4**	**5.(hexane)**	**6.(hexane)**
Formula	C52 H67 Al Be N2 O	C52 H67 Be Ga N2 O	C59 H81 Al Be N4 O (C6 H14)	C57 H84 Ga N5 O (C6 H14)
Fw (g mol^{-1})	772.06	814.80	984.43	1011.18
Cell setting	monoclinic	monoclinic	monoclinic	monoclinic
Space group	$P2_1/c$	$P2_1/c$	$P2_1/c$	$P2_1/c$
a (Å)	15.1705(18)	15.22540(10)	13.84490(10)	13.86510(10)
b (Å)	18.421(2)	18.47410(10)	21.1474(2)	21.4784(2)
c (Å)	17.1229(17)	17.15410(10)	20.3258(2)	20.0444(2)
α (°)	90	90	90	90
β (°)	105.781(9)	106.2730(10)	102.1830(10)	102.3790(10)
γ (°)	90	90	90	90
V (Å^{-3})	4604.8(9)	4631.72(5)	5817.03(9)	5830.45(9)
Z	4	4	4	4
ρ_{calc} (g cm^{-3})	1.114	1.168	1.124	1.152
Radiation, λ (Å)	1.54178	1.54184	1.54184	1.54184
Absorption	Multi-scan	Gaussian	Multi-scan	Multi-scan
μ (mm^{-1})	0.661	1.086	0.628	0.966
$R_{(int)}$	0.1135	0.0349	0.0598	0.0567
Parameters	530	530	728	823
R_1 (all data/I >2σ(I))	0.0581	0.037	0.0465	0.0660
ωR_2 (all data/I >2σ(I))	0.1635	0.1095	0.1229	0.1479
GooF	0.921	1.040	1.054	1.112
T (K)	100.0(2)	150.0(2)	100.0(2)	100.0(2)
CCDC Deposition No.	2235387	2235388	2235389	2235390

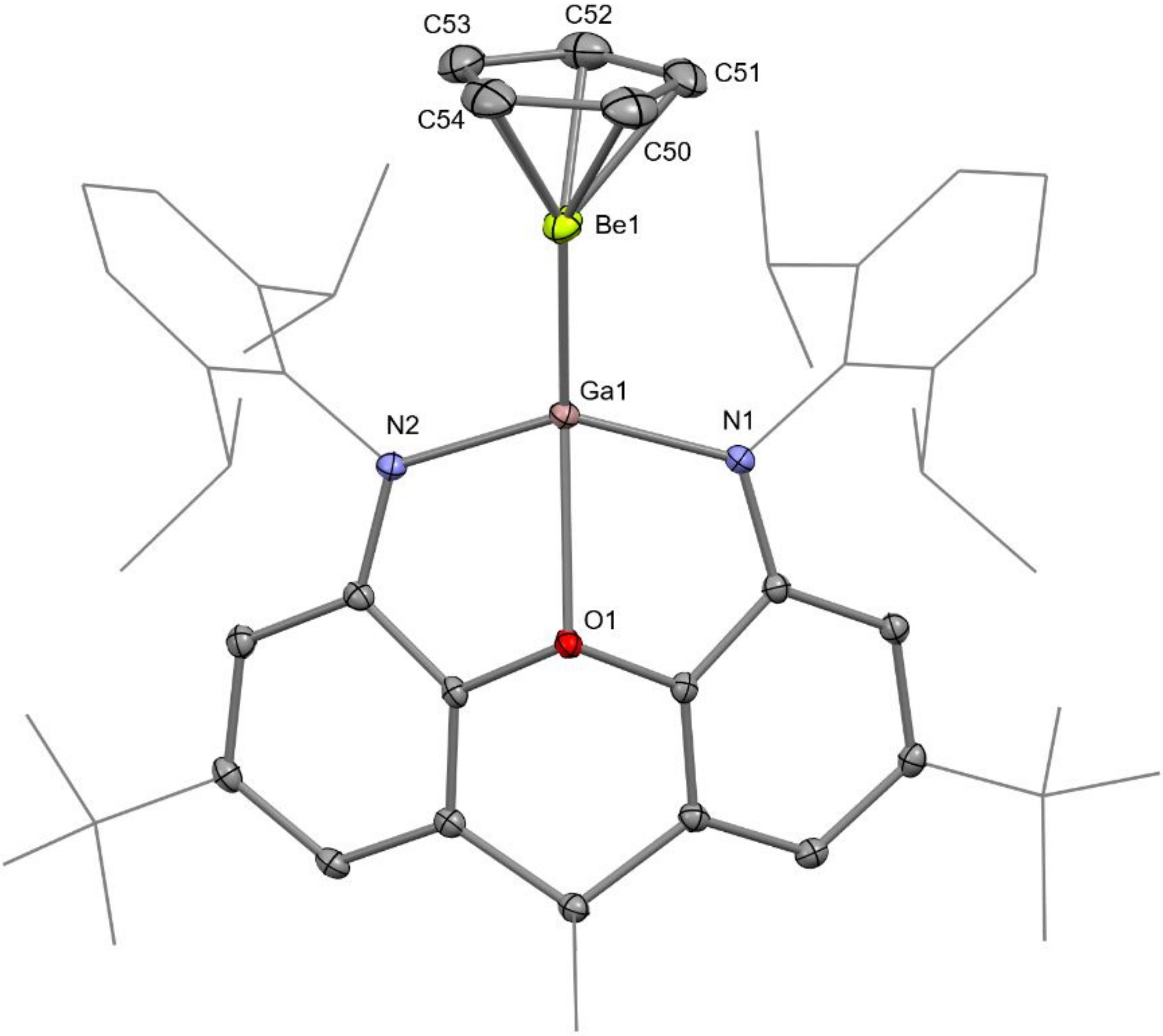

Figure S10: Molecular structure of **4** in the solid state as determined by X-ray crystallography. Thermal ellipsoids set at 50% probability; hydrogen atoms omitted, and selected substituents shown in wireframe format, for clarity.

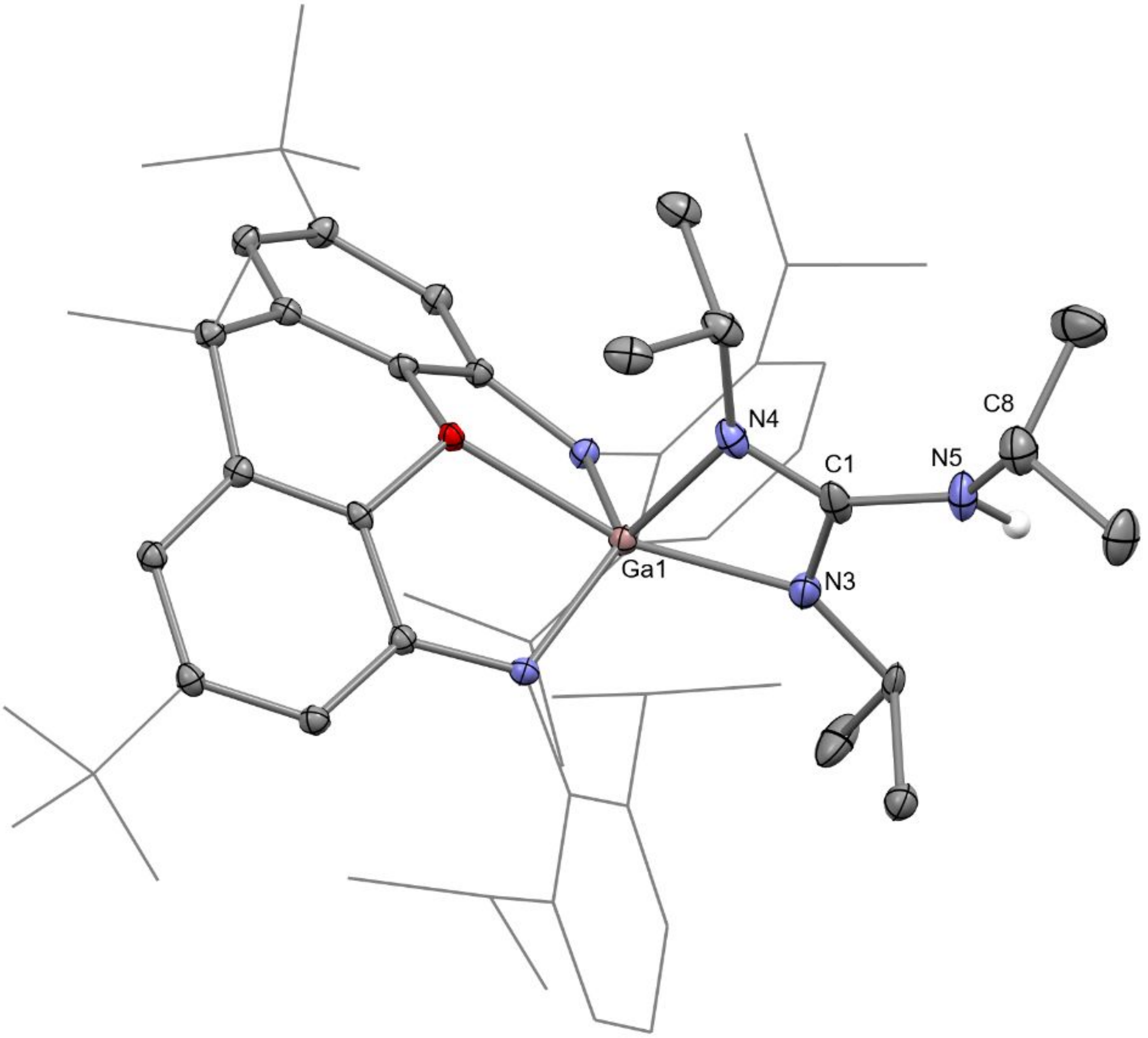

Figure S11: Molecular structure of **6** in the solid state as determined by X-ray crystallography. Thermal ellipsoids set at 50% probability; selected hydrogen atoms omitted, and selected substituents shown in wireframe format, for clarity.

^{9}Be NMR Chemical Shifts – CpBeR Complexes

Complexes **3** and **4** have been characterized by a range of analytical techniques, including multinuclear NMR spectroscopy. Perhaps most informative are the ^{9}Be NMR shifts for these species. In the case of both **3** and **4**, ^{9}Be NMR resonances (–28.8 and –26.9 ppm, respectively) are extremely high-field shifted, as is common for cyclopentadienyl-beryllium species. This is generally ascribed to ring current effects and to high electron density at the cyclopentadienyl ring, given the covalency of the Be-(η^5-C_5H_5) interaction. The value of δ_{9Be} measured for **3** is the highest field of all yet-reported beryllium species, exceeding even that of the silyl complex CpBe[Si(CH_3)$_3$] (–27.7 ppm). Other relevant examples include CpBe(CH_3) and CpBeCl for which the measured values of δ_{9Be} are –20.5 and –19.5 ppm, respectively. In addition, beryllium-carbene complex **5**, reported here, exhibits a ^{9}Be NMR chemical shift of -24.6 ppm. In broader terms, the ^{9}Be NMR chemicals shifts in the series CpBeR (R = [(NON)Al]$^-$, [Si(CH_3)$_3$]$^-$, [(NON)Ga]$^-$, [(NON)Al(NCN)]$^-$, [CH_3]$^-$, Cl^-) appear to correlate with the σ-donor strength of the R-group, with the aluminyl metalloligand possessing the strongest donor capabilities on this basis (Figure S12).

Table S2: ^{9}Be NMR chemical shifts for a range of CpBeR species and the Pauling electronegativity of the atom from each group which is bonded to beryllium

R-Group (CpBeR)	δ_{9Be} / ppm	Pauling Electronegativity of Donor Atom
[(NON)Al]- (**3**)	-28.8	1.61
Me_3Si-	-27.7	1.90
[Me_2($SiMe_3$)Si]-	-27.2	1.90
[(NON)Ga]- (**4**)	-26.9	1.81
[(NON)Al(NCN)]- (**5**)	-24.6	2.55
Me-	-20.5	2.55
Cl-	-19.5	3.16
Br-	-19.5	2.96

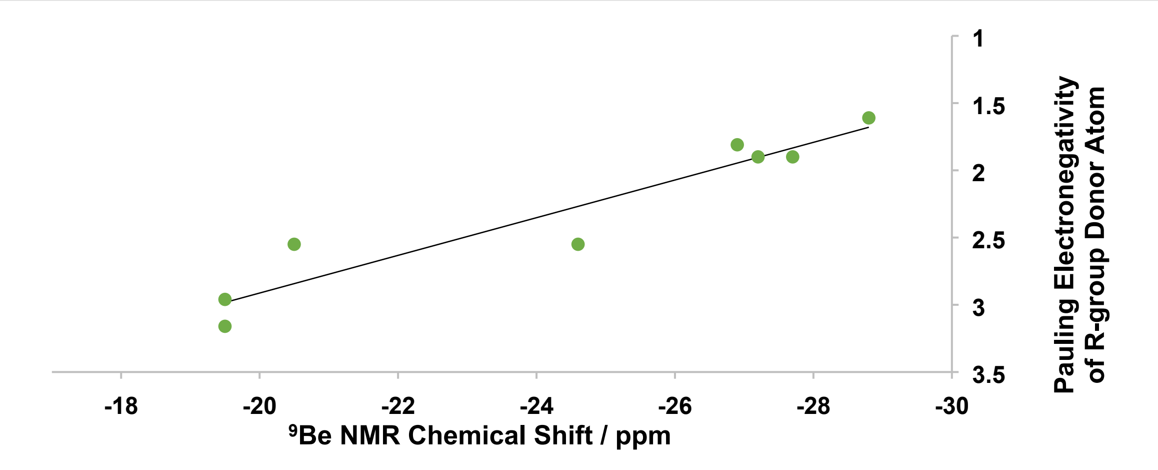

Figure S12: Plot of ^{9}Be NMR chemical shifts for a range of CpBeR species *vs* the Pauling electronegativity of the atom from each group which is bonded to beryllium.

Computational Details

The geometry optimizations (B3LYP D3BJ def2-TZVP def2/J) were performed with the ORCA (Revision 5.0.2) programme.[8-12] D3BJ dispersion corrections were used.[11,12] Full analytical frequency calculations were performed for the optimized structures to ensure the nature of the stationary points found (minima, no imaginary frequencies). The bonding and charge distribution in the complexes was examined by using Natural Atomic Orbital and Natural Bond Orbital analysis, in addition to Natural Population Analysis, as implemented in NBO7.[13] Topological and charge distribution analysis was carried out *via* QTAIM analysis (performed using the programme Multiwfn).[14] Molecular orbital diagrams are drawn with an isovalue of 0.05 a.u. Atom colours: pink, Al or Ga; green, Be; blue, N; red, O; grey, C. Hydrogen atoms omitted for clarity. In topological plots, blue points represent (3,-3) points/non-nuclear attractors and orange points represent bond critical points.

To probe the electronic structure of **3** and **4**, these compounds were investigated *via* quantum chemical calculations, as was the previously reported complex [(^{Dipp}Nacnac)(Br)AlBe(Br)(tmeda)] (**F**), for comparative purposes. The optimized structures of **3**, **4**, and **F** (B3LYP D3BJ def2-TZVP def2/J) agree satisfactorily with the molecular metrics determined by X-ray crystallography. The HOMO-LUMO gaps calculated for **3** and **4** are 4.56 and 4.13 eV, respectively. For **3**, the HOMO and HOMO-2 represent orbitals with Be–Al σ-bonding character (Figure S14). In the case of **4**, the HOMO and HOMO–4 are of Be–Ga σ-bonding character (Figure S16). In both **3** and **4** the LUMO comprises a Be–Al (Figure S13) or Be–Ga (Figure S15) in-phase combination of π-symmetry orbitals.

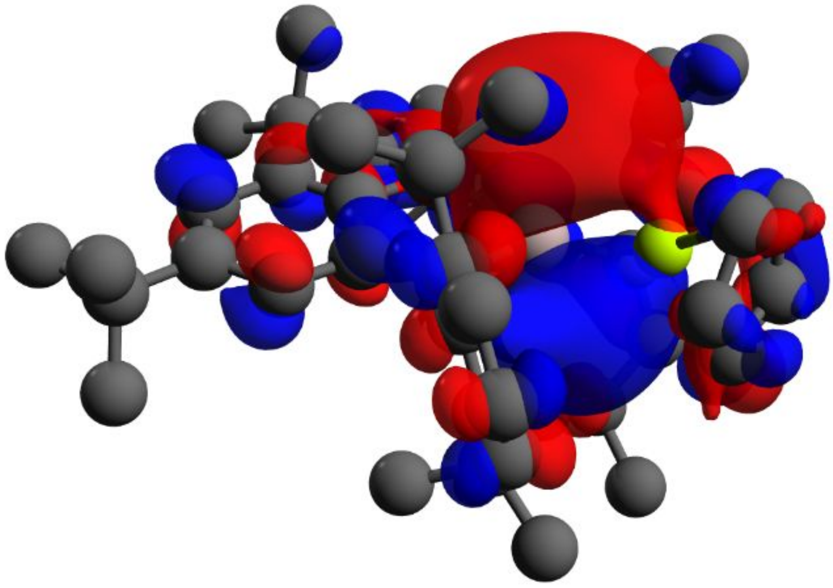

Figure S13: LUMO of compound **3**.

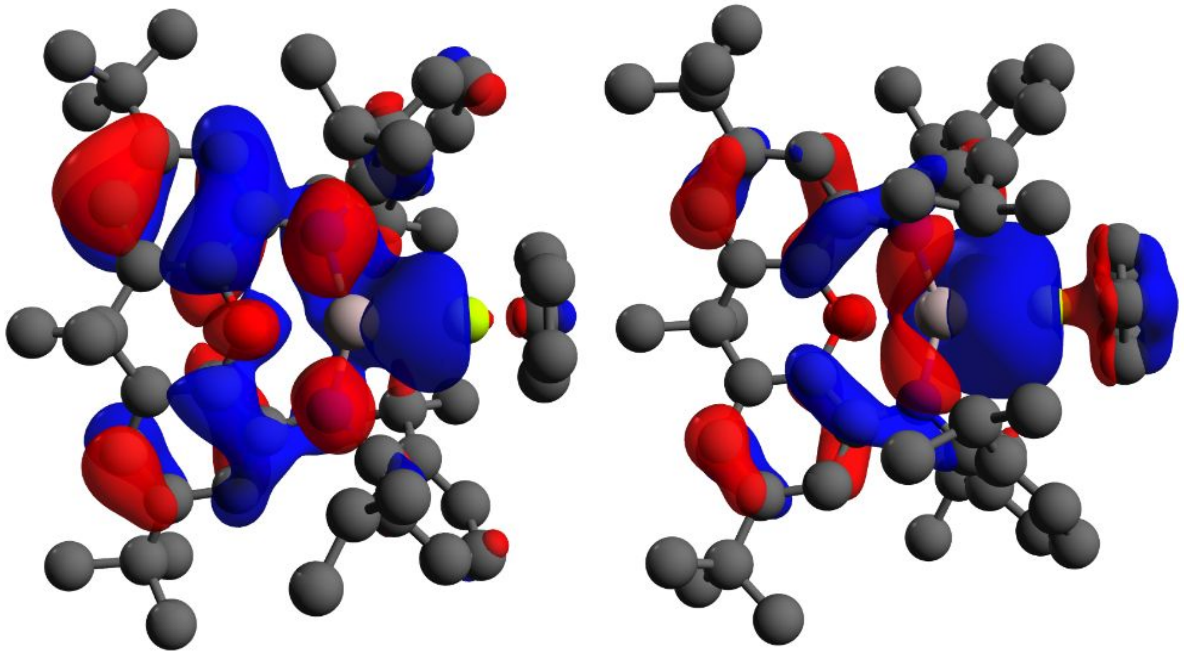

Figure S14: HOMO (left) and HOMO-2 (right) of compound **3**.

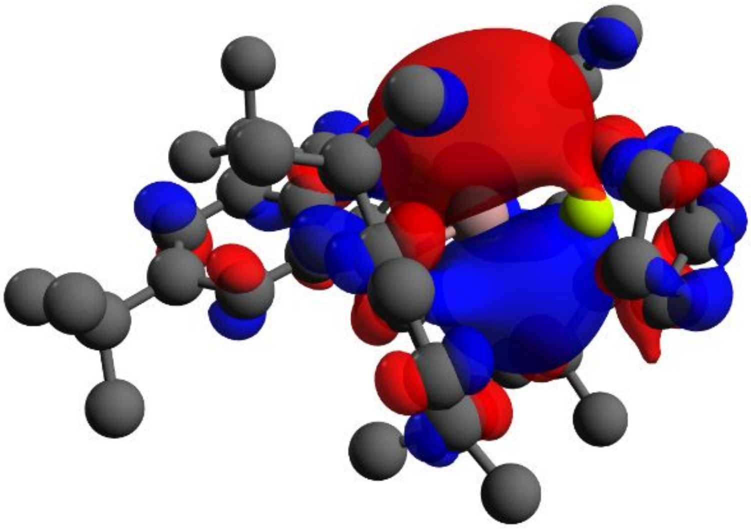

Figure S15: LUMO of compound **4**.

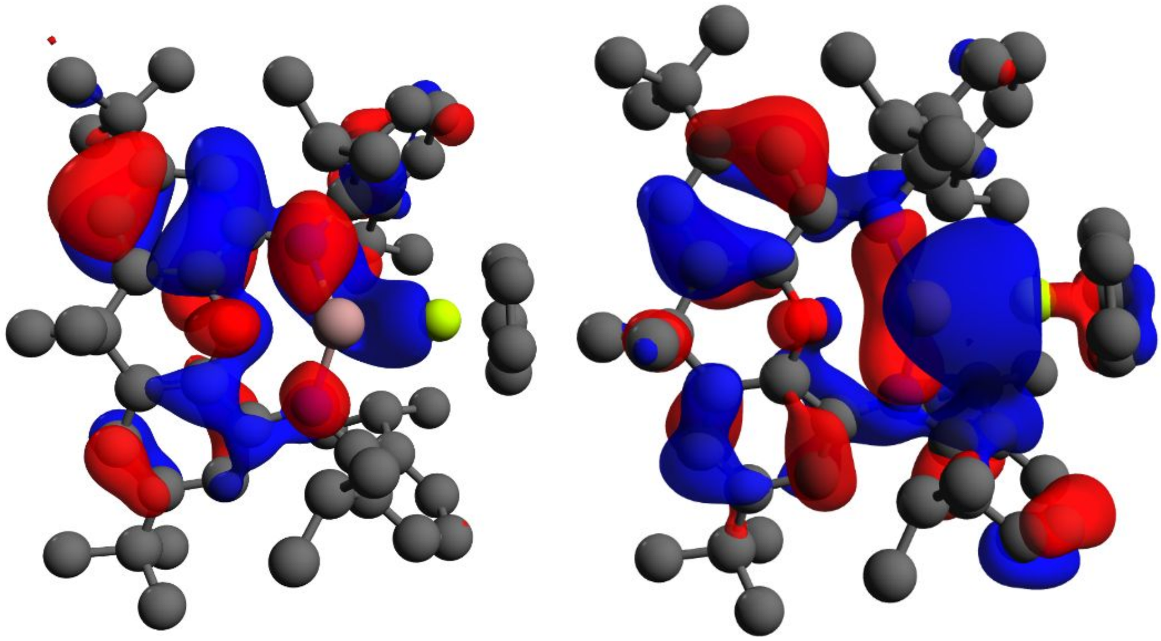

Figure S16: HOMO (left) and HOMO-4 (right) of compound **4**.

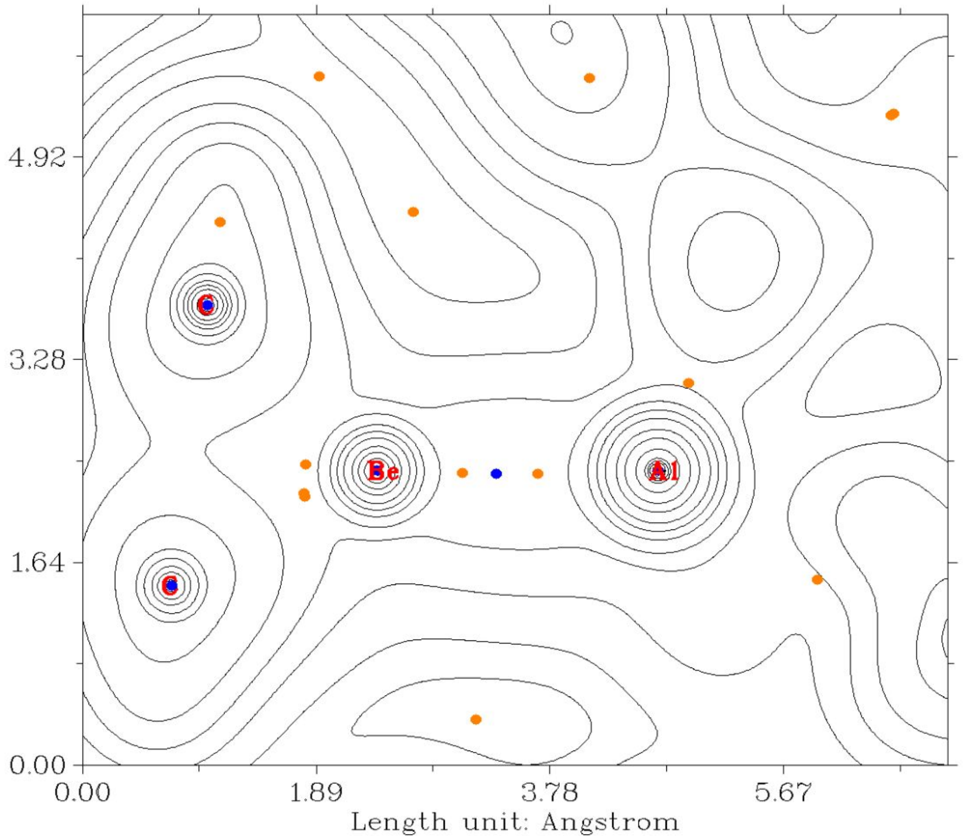

Figure S17: QTAIM derived plot of $\rho(r)$ for compound **3**.

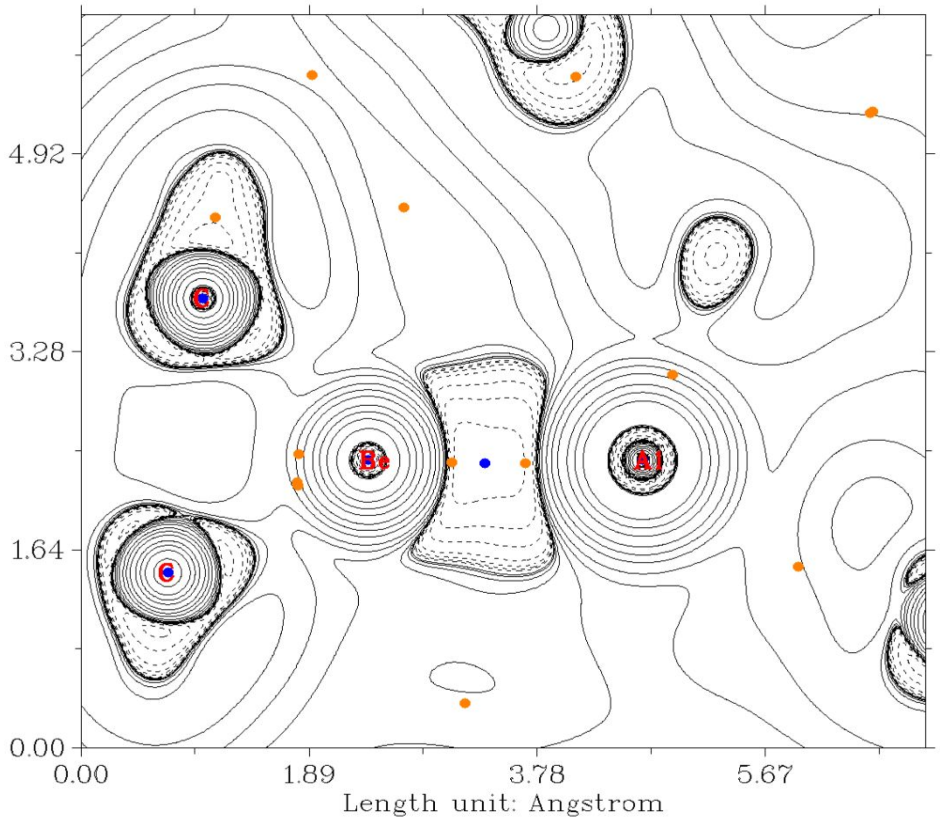

Figure S18: QTAIM derived plot of $\nabla^2\rho(r)$ for compound **3**.

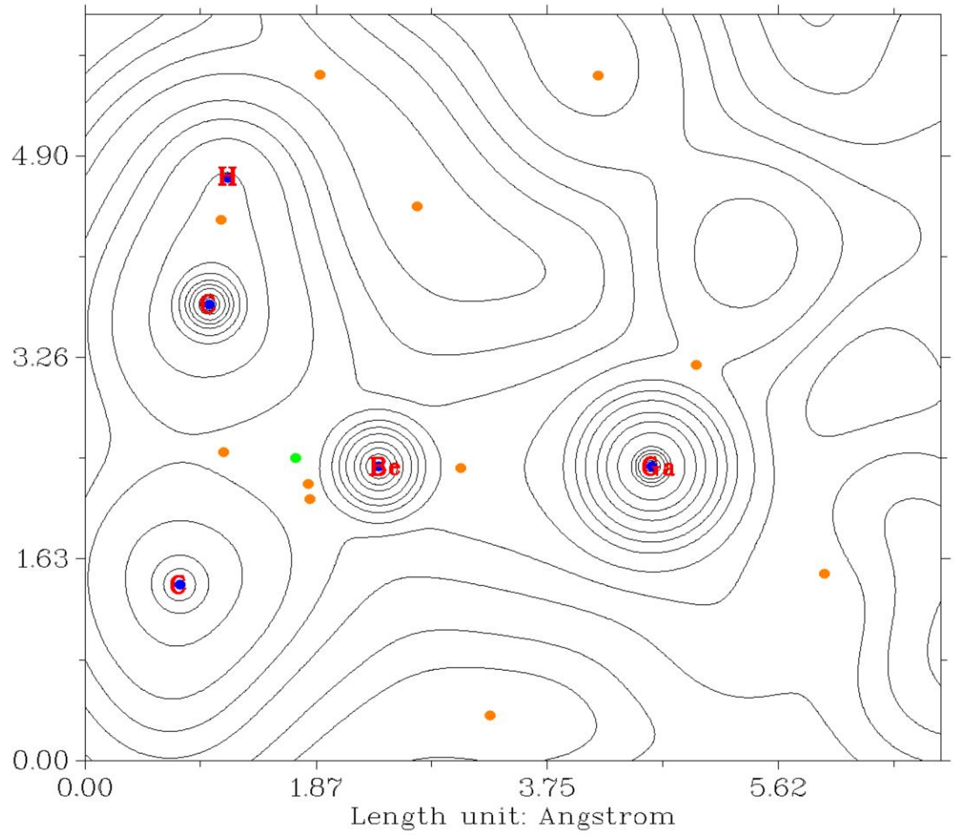

Figure S19: QTAIM derived plot of ρ(r) for compound **4**.

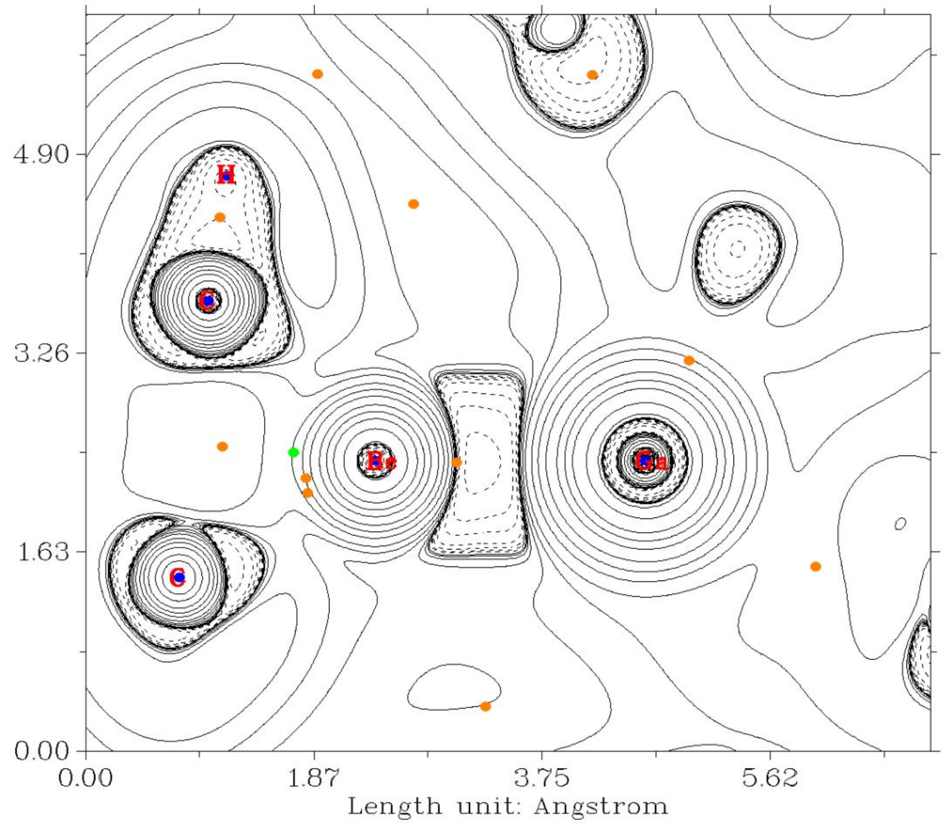

Figure S20: QTAIM derived plot of $\nabla^2\rho$(r) for compound **4**.

QTAIM Basin Analysis Charges

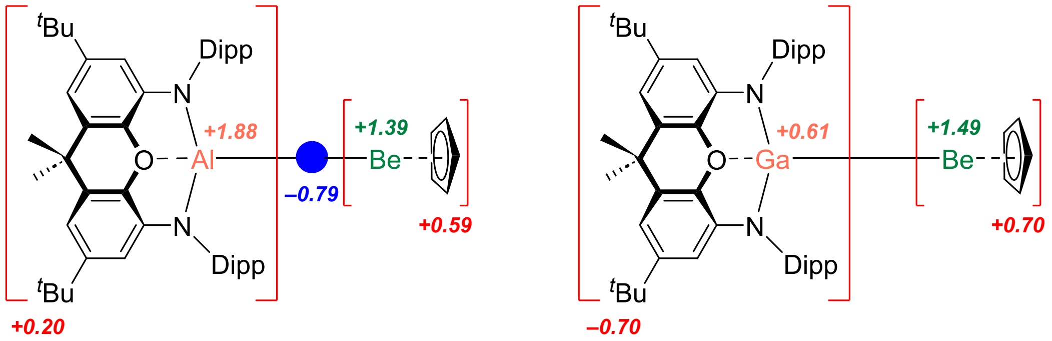

Figure S21: Pictorial summary of charge distribution within **3** (left) and **4** (right) as calculated using QTAIM basin analysis. The blue point is the (3,-3) non-nuclear attractor. Red values show the overall charge for the respective [(NON)E] and [BeCp] fragments.

NPA Charges

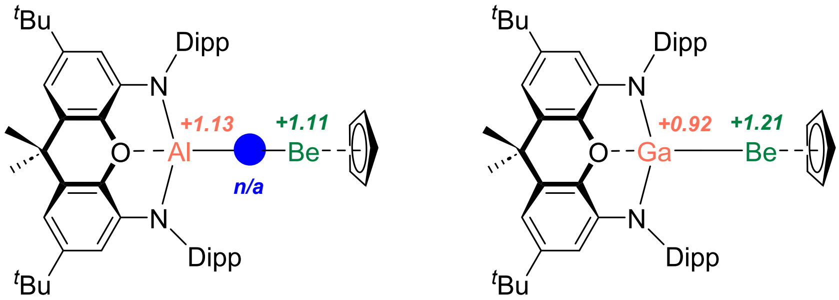

Figure S22: Pictorial summary of charge distribution within **3** (left) and **4** (right) as calculated using Natural Population Analysis. The blue point is the (3,-3) non-nuclear attractor, which NPA charges do not take into account.

For **3**, QTAIM basin analysis yields charges of +1.88, +1.39, and –0.79 for Al, Be, and the NNA, respectively, indicating that the charge at Al is higher than that at Be. Charges derived *via* Natural Population Analysis (NPA) of **3**, which do not take into account an NNA, suggest that the Be–Al bond is essentially homopolar, with charges of +1.13 for Al and +1.11 for Be. Both of these pictures of the charge distribution in **3** contrast with those calculated for **A**, **F**, and **G**, for which the charge at Al is meaningfully lower than that at Be. Within **4**, the QTAIM charge at Ga is calculated to be +0.61 and that at Be is +1.49. These values agree with those derived by NPA (Ga: +0.92; Be: +1.21); and thus, the polarity of the Be–Ga bond in **4** is opposite that of the Be–Al bond in **3**.

QTAIM Topological Analysis

Electron Density (top) and Laplacian of Electron Density (bottom)

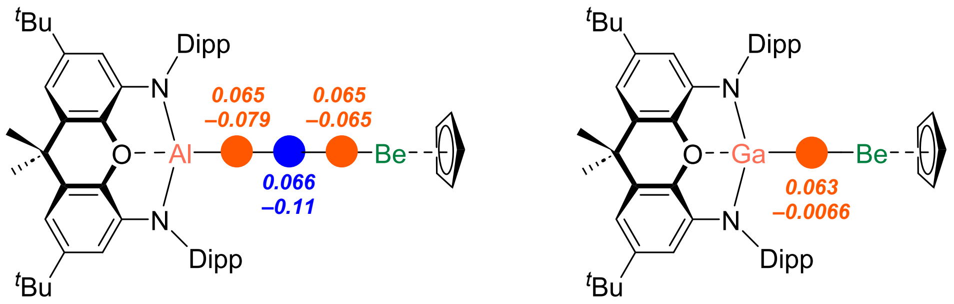

Figure S23: Pictorial summary of electron density *(ρ(r)*; e Å^{-3}; top)/Laplacian of electron density ($\nabla^2 \rho(r)$; e Å^{-5}; bottom) at the (3,-3) non-nuclear attractor (blue) or bond critical points (orange) within **3** (left) and **4** (right) as calculated using QTAIM Analysis.

Within **4**, a bond path is found between Be and Ga, with the electron density bond critical point (BCP) of 0.063 e Å^{-3}; typical of such an interaction. Within **3**, the electron density at the NNA (0.066 e Å^{-3}), and at the BCPs for the Be–NNA (0.065 e Å^{-3}) and Al–NNA (0.065 e Å^{-3}) interactions are all near-identical. This indicates that, topologically, the electron density in this region has a very flat profile in **3**, as is also observed for **H** and **I**. Moreover, the Laplacian of electron density at the NNA, Be–NNA BCP and Al–NNA (–0.11, –0.065, and –0.079 e Å^{-5}, respectively) are all characteristic of covalent bonding between the Be and Al centres in **3**, and are comparable to the related metrics for **I**. Analogous calculations performed on **F** do not indicate the presence of a NNA in this compound, presumably due to the greater Be–Al separation compared with **3**.

Table 3: xyz-coordinates of optimized structures

(NON)AlBeCp **3**				(NON)GaBeCp **4**			
Al	5.26394	5.13325	8.33607	Ga	5.21155	5.01488	8.25105
O	5.84391	6.88496	9.29117	O	5.83474	6.97423	9.27862
N	4.11298	5.13561	9.85930	N	4.10291	5.14584	9.87491
N	7.15432	5.08996	8.07966	N	7.17006	5.10074	8.07882
Be	4.28660	3.92064	6.68004	Be	4.27723	3.78402	6.66973
C	5.20663	7.09470	10.52102	C	5.20780	7.12966	10.50876
C	7.23248	7.04885	9.36535	C	7.21636	7.08669	9.35914
C	2.96443	4.29772	9.91236	C	2.95009	4.32147	9.92264
C	4.24014	6.13872	10.80142	C	4.25096	6.16176	10.79465
C	7.73947	4.31228	7.03961	C	7.75324	4.33176	7.03782
C	7.93511	6.06741	8.67184	C	7.91959	6.09518	8.67522
C	2.77728	3.51924	5.58127	C	2.77231	3.41929	5.58355
C	2.92335	2.56873	6.61907	C	2.91723	2.45764	6.61302
C	4.22028	2.00914	6.51309	C	4.21199	1.89197	6.49690
C	4.87672	2.60942	5.41069	C	4.86902	2.50130	5.39848
C	3.98545	3.54547	4.83469	C	3.97921	3.44724	4.83340
C	5.63448	8.08678	11.37230	C	5.63562	8.10867	11.37975
C	7.77933	8.03629	10.14531	C	7.78011	8.06063	10.14941
C	3.09460	2.95733	10.32893	C	3.07218	2.97459	10.32515
C	1.71447	4.79863	9.49969	C	1.70476	4.82870	9.50156
C	3.54534	6.30220	12.00724	C	3.56468	6.31424	12.00880
C	7.99823	2.94055	7.23905	C	8.01234	2.95835	7.23559
C	8.01052	4.91407	5.79469	C	8.00702	4.92953	5.78668
C	9.32562	6.18865	8.68529	C	9.31237	6.20779	8.69842
H	1.92150	4.15293	5.42245	H	1.92085	4.06191	5.43710
H	2.21562	2.38141	7.40970	H	2.21283	2.27050	7.40698
H	4.66466	1.31534	7.20672	H	4.65592	1.19073	7.18320
H	5.90564	2.45816	5.12925	H	5.89792	2.35153	5.11500
H	4.20844	4.20510	4.01310	H	4.20466	4.11645	4.02025
C	4.94411	8.19214	12.58117	C	4.96012	8.19837	12.59663
C	6.81873	8.95167	10.91810	C	6.82006	8.97591	10.92577
C	9.17892	8.10344	10.15882	C	9.17852	8.11452	10.17792
C	1.96909	2.13615	10.30607	C	1.94243	2.15969	10.29236
C	4.42676	2.43448	10.83675	C	4.40285	2.44043	10.82600
C	0.61525	3.94151	9.49563	C	0.60094	3.97786	9.48833
C	1.52777	6.24244	9.06544	C	1.53596	6.27123	9.05780
H	2.76451	5.59861	12.24690	H	2.78866	5.60452	12.24694
C	3.88684	7.32489	12.89415	C	3.90496	7.32738	12.90522
C	8.53435	2.20094	6.18620	C	8.53728	2.21719	6.17829
C	7.70235	2.28654	8.57765	C	7.73335	2.30697	8.57971
C	8.54781	4.13489	4.77152	C	8.53502	4.14916	4.75978
C	7.70606	6.37736	5.52084	C	7.68826	6.38952	5.51440
H	9.91159	5.46225	8.14096	H	9.89485	5.47557	8.15778
C	9.95268	7.20337	9.42109	C	9.94771	7.21231	9.43840
H	5.23521	8.95105	13.29101	H	5.25841	8.94688	13.31473
C	6.29394	10.04334	9.95540	C	6.29432	10.06984	9.96613
C	7.51157	9.62446	12.10335	C	7.51387	9.64694	12.11107
H	9.65925	8.85949	10.75696	H	9.66200	8.86015	10.78683
H	2.05073	1.10411	10.61710	H	2.01773	1.12478	10.59560
C	0.73608	2.61927	9.89087	C	0.71288	2.65279	9.87769
H	5.20927	2.92105	10.25295	H	5.18692	2.92345	10.24114
C	4.60020	0.92524	10.67265	C	4.56615	0.93063	10.65619
C	4.65305	2.84185	12.30040	C	4.63857	2.84389	12.28912
H	-0.34889	4.31532	9.17379	H	-0.35959	4.35824	9.16318
H	2.48984	6.74400	9.14570	H	2.50192	6.76371	9.14563
C	1.08626	6.34527	7.60116	C	1.11348	6.36473	7.58682
C	0.55088	6.98115	9.98971	C	0.55590	7.02617	9.96472
C	3.15389	7.49888	14.23097	C	3.17415	7.48707	14.24486
H	8.73830	1.14829	6.31827	H	8.74394	1.16492	6.31019
C	8.81198	2.78895	4.96033	C	8.80284	2.80363	4.94867
H	6.72400	2.65234	8.89971	H	6.76027	2.67228	8.91757
C	7.61650	0.76164	8.51243	C	7.64632	0.78188	8.52013
C	8.71747	2.70885	9.64911	C	8.76125	2.73445	9.63675
H	8.75503	4.58864	3.81044	H	8.73205	4.60124	3.79566
H	7.36849	6.83247	6.44861	H	7.34675	6.83958	6.44315
C	6.56384	6.52342	4.50747	C	6.54404	6.52439	4.50112
C	8.95269	7.14866	5.07194	C	8.92705	7.17263	5.06452
C	11.48594	7.26081	9.42346	C	11.48140	7.25464	9.44470
H	5.58465	10.69103	10.47385	H	5.58576	10.71665	10.48697
H	5.78813	9.60098	9.09713	H	5.78794	9.62880	9.10780
H	7.12325	10.65086	9.58846	H	7.12343	10.67839	9.60008
H	8.33636	10.24907	11.76047	H	8.33844	10.27228	11.76853
H	7.90248	8.88936	12.80744	H	7.90578	8.91092	12.81380

H	6.81490	10.27464	12.63241	H	6.81752	10.29605	12.64218
H	-0.12709	1.96555	9.87937	H	-0.15418	2.00432	9.86055
H	5.61429	0.63699	10.95093	H	5.58010	0.63500	10.92731
H	4.42779	0.60779	9.64287	H	4.38650	0.61695	9.62623
H	3.91965	0.36522	11.31709	H	3.88552	0.37231	11.30210
H	3.88060	2.40702	12.93988	H	3.86501	2.41535	12.93150
H	4.62516	3.92283	12.42111	H	4.62103	3.92506	12.40967
H	5.62514	2.48624	12.65020	H	5.60901	2.47956	12.63443
H	1.82913	5.89379	6.94410	H	1.85601	5.89345	6.94213
H	0.96717	7.39199	7.31204	H	1.01513	7.40956	7.28370
H	0.13161	5.84156	7.43308	H	0.15312	5.87423	7.41215
H	-0.45228	6.55259	9.93360	H	-0.44998	6.60447	9.90409
H	0.48172	8.03377	9.70662	H	0.49750	8.07643	9.67082
H	0.88357	6.93148	11.02666	H	0.88000	6.98421	11.00484
C	2.00192	6.50112	14.40221	C	2.02653	6.48356	14.41035
C	2.56828	8.92067	14.31614	C	2.58267	8.90589	14.34113
C	4.14929	7.28232	15.38680	C	4.17139	7.26685	15.39817
H	9.22841	2.19592	4.15572	H	9.21279	2.20976	4.14132
H	8.58410	0.31354	8.27702	H	8.61137	0.33195	8.27778
H	6.89869	0.42456	7.76254	H	6.92186	0.44184	7.77773
H	7.30587	0.36614	9.47902	H	7.34355	0.39009	9.49095
H	8.47120	2.24824	10.60888	H	8.53230	2.26991	10.59882
H	8.73363	3.78740	9.78493	H	8.76898	3.81277	9.77438
H	9.72245	2.38530	9.36703	H	9.76474	2.42008	9.33931
H	6.82413	6.07413	3.54628	H	6.81053	6.08504	3.53707
H	6.33769	7.57839	4.33570	H	6.30134	7.57659	4.33582
H	5.66060	6.03630	4.87616	H	5.64644	6.02156	4.86417
H	9.33055	6.78326	4.11451	H	9.31078	6.80693	4.10938
H	9.75137	7.05835	5.80806	H	9.72447	7.09301	5.80331
H	8.71933	8.20906	4.95358	H	8.68331	8.23015	4.94187
C	12.03942	5.96393	10.04410	C	12.02283	5.94808	10.05554
C	11.99783	7.39319	7.97731	C	11.99705	7.39388	8.00043
C	12.02862	8.44711	10.22961	C	12.03322	8.42939	10.26126
H	1.50552	6.67737	15.35829	H	1.53144	6.64964	15.36905
H	2.35503	5.46862	14.39955	H	2.38345	5.45243	14.39763
H	1.25308	6.60746	13.61534	H	1.27555	6.59399	13.62595
H	2.03148	9.05348	15.25855	H	2.04777	9.03119	15.28579
H	1.86975	9.10022	13.49658	H	1.88147	9.08758	13.52424
H	3.34705	9.68188	14.26456	H	3.35872	9.66997	14.29135
H	4.96970	7.99942	15.34956	H	4.98959	7.98655	15.36399
H	4.57849	6.27973	15.34048	H	4.60381	6.26591	15.34553
H	3.64505	7.39463	16.34966	H	3.66836	7.37226	16.36258
H	11.72777	5.08504	9.47946	H	11.70412	5.07649	9.48347
H	11.68388	5.84534	11.06944	H	11.66451	5.82439	11.07932
H	13.13177	5.98574	10.05993	H	13.11542	5.95971	10.07321
H	13.08903	7.44617	7.96471	H	13.08897	7.43355	7.98880
H	11.60556	8.29885	7.51082	H	11.61614	8.30852	7.54199
H	11.69934	6.54339	7.36338	H	11.68801	6.55335	7.37882
H	13.11917	8.44905	10.18497	H	13.12397	8.42169	10.21977
H	11.74165	8.38792	11.28095	H	11.74227	8.36485	11.31120
H	11.67720	9.40037	9.83037	H	11.69147	9.38886	9.86856

NacNacAl(Br)Be(Br)(TMEDA) **F**

Br	10.63647	15.49850	2.15298
Al	11.25919	15.49825	4.52788
N	10.08371	14.06124	5.14233
C	7.85089	13.07196	5.51700
H	7.39492	12.78176	4.56885
H	7.04893	13.35204	6.19759
H	8.37265	12.20777	5.91784
C	8.77436	14.24203	5.28633
C	8.16399	15.49913	5.26015
H	7.08955	15.49943	5.36820
C	10.66647	12.76059	5.33661
C	11.28691	12.48920	6.57753
C	11.90747	11.25506	6.75194
H	12.38939	11.03010	7.69185
C	11.93267	10.31013	5.73633
H	12.43089	9.36101	5.88979
C	11.31011	10.58394	4.53297
H	11.31363	9.83642	3.75035
C	10.65210	11.79613	4.31034
C	11.26007	13.50086	7.71312
H	11.47162	14.47677	7.27363
C	12.32291	13.25212	8.78401
H	12.09382	12.36434	9.37936
H	12.35466	14.10425	9.46394
H	13.31585	13.14577	8.35238
C	9.87984	13.57211	8.38558

H	9.10697	13.93613	7.71431
H	9.91865	14.25135	9.23910
H	9.58253	12.58700	8.75336
C	9.88837	11.96580	3.00482
H	9.43753	12.95633	2.99622
C	8.77129	10.91416	2.88837
H	9.18898	9.91725	2.73168
H	8.13074	11.14239	2.03392
H	8.14880	10.87061	3.78104
C	10.78872	11.87365	1.76722
H	11.50687	12.68796	1.73747
H	10.18270	11.94858	0.86229
H	11.32571	10.92306	1.73424
N	10.08451	16.93598	5.14244
C	7.85216	17.92644	5.51701
H	7.39610	18.21663	4.56890
H	7.05021	17.64685	6.19782
H	8.37439	18.79048	5.91755
C	8.77505	16.75590	5.28640
C	10.66799	18.23636	5.33670
C	11.28874	18.50751	6.57756
C	11.91011	19.74127	6.75173
H	12.39243	19.96597	7.69150
C	11.93572	20.68612	5.73606
H	12.43460	21.63492	5.88934
C	11.31290	20.41256	4.53279
H	11.31683	21.16000	3.75008
C	10.65416	19.20075	4.31032
C	11.26195	17.49591	7.71324
H	11.47275	16.51984	7.27372
C	12.32554	17.74425	8.78346
H	12.09727	18.63227	9.37876
H	12.35723	16.89224	9.46354
H	13.31829	17.84999	8.35123
C	9.88216	17.42545	8.38667
H	9.10863	17.06176	7.71603
H	9.92121	16.74631	9.24026
H	9.58564	18.41076	8.75455
C	9.89047	19.03143	3.00474
H	9.43865	18.04135	2.99631
C	8.77444	20.08416	2.88781
H	9.19316	21.08059	2.73083
H	8.13376	19.85628	2.03336
H	8.15190	20.12863	3.78038
C	10.79110	19.12242	1.76728
H	11.50856	18.30750	1.73791
H	10.18515	19.04773	0.86227
H	11.32889	20.07255	1.73406
Be	13.59944	15.49728	4.87402
Br	14.40521	15.49603	6.94381
N	14.53258	16.84088	4.06157
C	15.90159	16.27026	3.90875
H	16.48844	16.60669	4.75872
H	16.36469	16.66848	3.00299
C	13.96779	17.22757	2.74933
H	14.58000	18.01338	2.29583
H	12.96088	17.59715	2.90111
H	13.91549	16.38533	2.07209
C	14.57818	18.08460	4.87091
H	14.97177	17.86302	5.85644
H	13.56904	18.47233	4.97451
H	15.20012	18.83328	4.37068
N	14.53178	14.15369	4.06050
C	15.90105	14.72379	3.90730
H	16.48861	14.38544	4.75602
H	16.36294	14.32690	3.00036
C	13.96606	13.76795	2.74838
H	14.57796	12.98251	2.29383
H	12.95931	13.39815	2.90068
H	13.91311	14.61070	2.07183
C	14.57723	12.90951	4.86915
H	14.97146	13.13037	5.85460
H	13.56797	12.52217	4.97309
H	15.19856	12.16080	4.36819

References

1. Hicks, J.; Vasko, P.; Goicoechea, J. M.; Aldridge, S. Synthesis, structure and reaction chemistry of a nucleophilic aluminyl anion. *Nature* **2018**, *557*, 92.
2. Fischer, E. O.; Hofmann, H. P., Über Aromatenkomplexe von Metallen, XXV. Di-cyclopentadienyl-beryllium. *Chem. Ber.* **1959**, *92*, 482.
3. Cosier, J.; Glazer, A. M. A nitrogen-gas-stream cryostat for general X-ray diffraction studies. *J. Appl. Cryst.* **1986**, *19*, 105.
4. CrysAlisPro, Agilent Technologies, Version 1.171.39.46
5. Sheldrick, G. M. SHELXT - Integrated space-group and crystal-structure determination. *Acta Crystallogr., Sect. A: Found. Adv.* **2015**, *71*, 3.
6. Sheldrick, G. M. Crystal structure refinement with SHELXL. *Acta Crystallogr., Sect. C: Struct. Chem.* **2015**, *71*, 3.
7. Dolomanov, O. V.; Bourhis, L. J.; Gildea, R. J.; Howard, J. A. K.; Puschmann, H. OLEX2: a complete structure solution, refinement and analysis program. *J. Appl. Cryst*. **2009**, *42*, 339.
8. Neese, F. The ORCA program system, *Wiley Interdiscip. Rev.: Comput. Mol. Sci*. **2012**, *2*, 73.
9. Neese, F. Software update: the ORCA program system, version 4.0, *Wiley Interdiscip. Rev.: Comput. Mol. Sci.* **2017**, *8*, e1327.
10. Neese, F.; Wennmohs, F.; Becker, U.; Riplinger, C. The ORCA quantum chemistry program package, *J. Chem. Phys.* **2020**, *152*, 224108.
11. Grimme, S.; Ehrlich, S.; Goerigk, L. Effect of the damping function in dispersion corrected density functional theory. *J. Comput. Chem.* **2011**, *32*, 1456.
12. Grimme, S. A generally applicable atomic-charge dependent London dispersion correction. *J. Chem. Phys.* **2019**, *150*, 154122.
13. SX NBO 7.0. Glendening, E. D.; Badenhoop, J. K.; Reed, A. E.; Carpenter, J. E.; Bohmann, J. A.; Morales, C. M.; Karafiloglou, P.; Landis, C. R.; Weinhold, F. Theoretical Chemistry Institute, University of Wisconsin, Madison, WI 2018.
14. Tian Lu, Feiwu Chen, Multiwfn: A Multifunctional Wavefunction Analyzer, *J. Comput. Chem.* **2012**, *33*, 580.
15. *X-Area 1.8.1*, STOE & Cie GmbH, Darmstadt, Germany, **2018**
